# Aloe vera-derived extracellular vesicle-like particles suppress pancreatic carcinoma progression through triggering pyroptosis via ROS-GSDMD/E signaling pathway

**DOI:** 10.1186/s13020-025-01153-7

**Published:** 2025-07-02

**Authors:** Jieyu Shen, Tianfu Wei, Mingchen Li, Yuankuan Jiang, Jiahui Zhang, Yueyi Qi, Cai Chen, Xiaojie Li, Peng Huang, Jialin Qu

**Affiliations:** 1 Clinical Laboratory of Integrative Medicine, The First Affiliated Hospital of Dalian Medical University, No. 222, Zhongshan Road, Dalian, 116011 China; 2 Institute of Integrative Medicine, Dalian Medical University, No. 9, South Road of Lvshun, Dalian, 116044 China; 3 Institute (College) of Pharmacy, Dalian Medical University, No. 9, South Road of Lvshun, Dalian, 116044 China

**Keywords:** Plant-derived extracellular vesicle-like particles, Aloe Vera, Pancreatic carcinoma, Pyroptosis

## Abstract

**Background:**

Pancreatic carcinoma (PC) remains one of the most aggressive malignancies that is often referred to as the “king of cancers” in clinic. Plant-derived extracellular vesicle-like particles (p-EVLP) has demonstrated broad-spectrum antitumor potential through their unique ability to effectively penetrate tumor microenvironments and deliver bioactive compounds. Aloe Vera is a tender and juicy plant with anti-tumor properties, while whether Aloe Vera-derived EVLP (AV-EVLP) can inhibit PC and what the underlying mechanism is still unclear.

**Methods:**

Two kinds of AV-EVLPs (EV-U and EV-P) were isolated from Aloe vera using comparative purification techniques. Their structure and composition characterization were performed using TEM, NTA and UHPLC-QTOFMS. In vitro experiments using Panc-1 cells included cytotoxicity, migration/invasion and cellular uptake assay were employed to investigate their tumor inhibition potential. In a Panc-1 xenograft mouse model, the therapeutic effects and systemic toxicity of EV-U were evaluated through tumor volume and weight, Ki67, TUNEL and histopathology examination. Mechanistic studies involved the levels of cellular ROS, IL-1β, IL-18 and the expression of caspase-1/3/7/9-GSDMD/E in both cell and tumor tissues were determined by ELISA, immunohistochemistry, Western blot and qRT-PCR.

**Results:**

EV-U and EV-P exhibited characteristic cup-shaped morphology with mean diameters 179.3 nm and 227.1 nm, respectively. At their respective IC_50_ concentrations, both effectively inhibit cell migration and invasion and increase ROS, LDH, IL-18, and IL-1β levels in Panc-1 cells. Comparably, EV-U exhibited better activity due to their fewer impurities and more uniform dispersion. Further in vivo experiments supported the effectiveness of EV-U in reducing tumor volume and weight without causing toxicity or immunogenicity. Mechanistically, the activation of pyroptosis through the caspase-1/3/7/9-GSDMD/E pathways contributed to its efficacy.

**Conclusion:**

AV-EVLP significantly inhibit pancreatic cancer progression by triggering mitochondrial ROS release through the activation of caspase-1/3/7/9-GSDMD/E-mediated pyroptosis.

**Graphic Abstract:**

AV-EVLPs effectively inhibited the migration and invasion of Panc-1 cell and reduced tumor volume and weight in Panc-1 tumor-bearing mice, whose mechanism was related to triggering pyroptosis via ROS-GSDMD/E pathways.

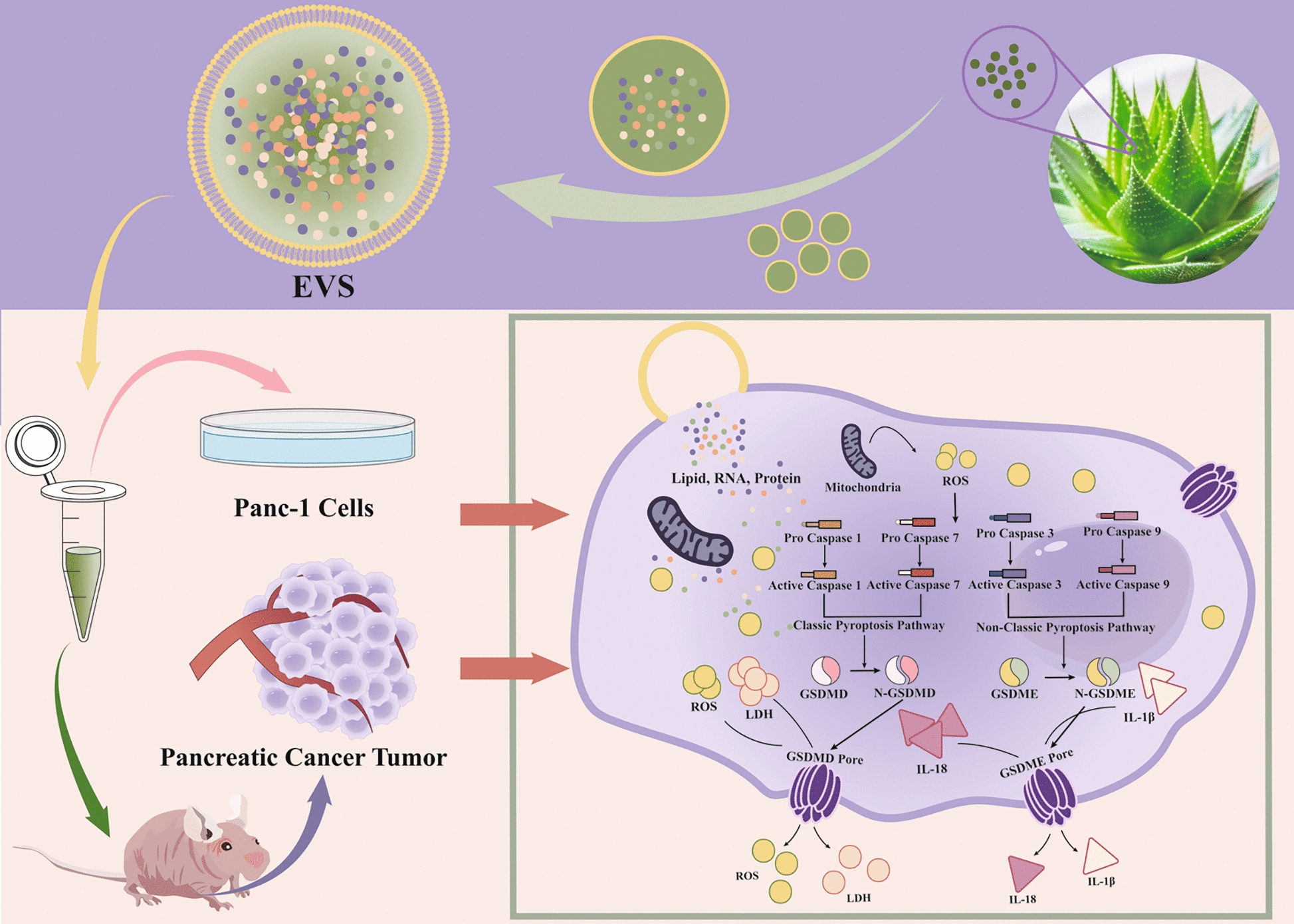

**Supplementary Information:**

The online version contains supplementary material available at 10.1186/s13020-025-01153-7.

## Highlights

- The extracellular vesicles-like particles isolated from Aloe Vera by ultracentrifugation (EV-U) demonstrated enhanced structural homogeneity and superior antitumor efficacy against Panc-1 cells compared to those extracted by polymer precipitation (EV-P)
- EV-U showed in vivo efficacy in a pancreatic cancer xenograft model characterized by reduced tumor volume and weight without causing toxicity or immunogenicity
- Gasdermin-mediated pyroptosis contributes to the protective effects of AV-EVLPs against pancreatic cancer progression and offer targets for further therapy

**Supplementary Information:**

The online version contains supplementary material available at 10.1186/s13020-025-01153-7.

## Introduction

Pancreatic carcinoma (PC), a highly malignant neoplasm of the digestive system, is projected to become the second leading cause of cancer-related mortality worldwide by 2030 [1]. As most patients are diagnosed with late-stage and lose the opportunity for surgery due to its atypical early symptoms and insidious progression, the 5-year survival rate for PC patients is only 10% according to a survey reported by Lancet [2]. Recently, immunotherapy strategies like targeting programmed cell death protein 1 (PD-1) and its ligand (PD-L1) have shown promise in various cancers [3]. However, their clinical efficacy in PC remains suboptimal, with both monotherapy and immune checkpoint inhibitor yielding disappointing outcomes than anticipated [4]. This therapeutic limitation may be attributed to the formation of a unique immune-suppressive tumor microenvironment (TME) that hides from the immune system and allows tumor growth and metastasis, which is enriched for cancer cells, immune cells, cancer-associated fibroblasts, and other stromal elements [5]. Besides, research has demonstrated that the intratumoral effector T cells present in pancreatic ductal adenocarcinoma (PDAC) are relatively scarce, particularly in the juxtatumoral stroma located in close proximity to the cancer cells. This has led to the designation of PDAC as one of the “coldest of the cold” tumors [6]. Consequently, strategies to remodel the TME and achieve successful targeted delivery of drugs to PC cells have emerged as a major focus of research [7, 8].

Pyroptosis, a form of inflammatory cell death that is triggered by Gasdermin proteins, releases a multitude of molecules that signal “find me” and present “eat me” signal. It exhibited potential against cancer via converting the immunosuppressive “cold” TME into immunogenic “hot” TME by recruiting lymphocytes [9]. Unlike necrosis, pyroptosis is triggered by inflammatory caspases in most cases, leading to the release of cytokines IL-1β and other IL-1 family members, which further activate T cell-mediated immune responses. This activation stimulates the host’s immune system and subsequently suppresses tumor proliferation and metastasis. Therefore, activating pyroptosis-related pathway provides a new perspective for the development and treatment of PC [10]. Recently, the efficacy of several chemotherapeutic agents (triptolide, topotecan, irinotecan, etc.) has been demonstrated in various cell model (Jurkat, MeWo, NCI-H522 and HeLa), whose pyroptosis-related mechanism were both associated with caspase-3 activation and subsequent induction of gasdermin D (GSDMD) [11, 12]. But poor water solubility and immunosuppressive effects limit their clinical translation. In contrast, nanomedicines could overcome above inferiority due to their diverse structural designs, targeted delivery systems as well as stable physical and chemical properties [13].

Recent studies have shown that nanomedicines are a promising alternative to chemotherapy for PDAC [14]. They accumulate more easily at the tumor site than their corresponding unpackaged drugs [15]. Additionally, they could reshape the TME and reverse tumor multidrug resistance by overcoming the ‘protective barrier’ [16]. For instance, gemcitabine-loaded nanoparticles exhibited superior antitumor efficacy and reduced systemic toxicity compared to free drug in a patient-derived xenograft model and Panc-1 cells [17]. Surface modification with tumor-homing peptides further enhances cellular uptake and cytotoxicity in Panc-1 cells [18]. However, clinical application and development of nano-preparations are limited due to high raw material demand, cost and biocompatibility [19]. Therefore, there is an urgent need to develop a new generation of nanomedicine characterized by safety, high efficacy, cost-effectiveness and suitability for large-scale production.

Plant-derived extracellular vesicle-like particles (p-EVLP) had drawn attention in recent decade in view of their natural abundance, exceptional biological effects, high yields and consistent properties [20]. Besides, similar or better biological functions with their homologous plants were found due to their enclosed constituents as well as specific lipid components, nucleic acids and proteins, which could realize the signal transmission between different species [21]. On the one side, EVLPs derived from ginger [22], tea [23], bitter melon [24] and other sources can be directly effective in treating various diseases (chronic colitis, breast cancer, oral squamous cell carcinoma, etc.) [25]. On the other hand, the encapsulation potential observed in grapefruit derived EVLPs due to their low toxicity and non-penetration of the placental barrier provide new ideas and methods for the development of safer and more effective drug delivery systems [26]. Besides, the plants that possess the characteristics of stability as well as unlimited yield of season were the reliable sources for the research subject for EVLPs.

Aloe Vera, also known as ‘Lu-Hui’ in Chinese, has been used for purging fire, detoxifying toxicosis, and dispersing blood stasis. Modern pharmacological studies have explored its broad-spectrum antitumor activities, with anthraquinones and polysaccharides (two major compositional classes) being systematically studied and widely recognized [27]. As a tender and juicy plant that possesses the characteristics of stability and season-independent yield, Aloe vera is a valuable source for researching p-EVLP. However, it remains unclear whether these Aloe vera-derived extracellular vesicle-like particles (AV-EVLP) possess the ability to inhibit PC, and their underlying mechanism has not yet been elucidated.

In the present research, EVLPs from Aloe Vera was comparatively isolated and purified by ultra-centrifugation and a proprietary polymer-based reagent (PEG-6000). Their structures and compositions were characterized via transmission electron microscopy (TEM), nanoparticle tracking analysis (NTA) and ultra-high-performance liquid chromatography-mass spectrometry (UHPLC-MS). Subsequently, their anti-proliferative activity against pancreatic carcinoma was evaluated using Panc-1 cells and tumor-bearing mice. Moreover, pyroptosis-associated mechanisms involving both classic and non-classic pathway were investigated. Our results not only enrich the understanding of pharmacodynamic material bases of Aloe Vera, but also provide insights into its therapeutic potential and mechanism against pancreatic carcinoma via activation of pyroptosis-associated inflammasome pathways.

## Materials and methods

### Chemicals, reagents, and materials

Fresh Aloe vera is purchased from the Plantation in Yuanjiang County (Yunnan Province, China) and authenticated as the whole plant of *Aloe vera* (L.) Burm. f. by Prof. Aijing Leng (Department of traditional Chinese medicine, The First Affiliated Hospital of Dalian Medical University).

The Amicon^®^ Ultra-4 centrifugal filter (100 kDa), PVDF membrane (0.22 μM), ultracentrifuge tubes, chamber (8 mm pore size) were supplied by Merck & Co., Inc. (Rahway, NJ, USA), Beckman Coulter Commercial Enterprise (China) Co. Ltd (Shanghai, China) and Corning (Shanghai) Co., Ltd (Shanghai, China), respectively. Biochemical reagents including 3,3′-dioctadecyloxacarbocyanine perchlorate (DIO), DAPI, brilliant blue R250, MTT, PEG-6000, Triton X-100, total protein extraction kit, LDH, ROS and BCA test kits were purchased from Beijing Solarbio Science & Technology Co., Ltd (Beijing, China). Matrigel (ABW) was purchased from Bitab Biotech Co., Ltd (Beijing, China). ELISA kits including terminal-deoxynucleotidyl transferase mediated nick end labeling (TUNEL), interleukin-18 (IL-18) and interleukin-1 beta (IL-1β) were purchased from ELabscience Biotechnology Co., Ltd (Beijing, China).

Rabbit monoclonal antibodies against GSDMD, caspase-1/3/7/9 and β-actin were obtained from AB clonal Biotechnology (Wuhan, Hubei, China). Additionally, rabbit monoclonal antibodies against GSDME were purchased from Hangzhou HuaAn Biotechnology Co., Ltd (Hangzhou, China). Trizol reagent, cDNA reverse transcription kits and primers were purchased from Accurate Biotechnology Co., Ltd (Changsha, Hunan, China).

### Isolation and purification of EVLPs from Aloe vera by differential ultra-centrifugation and PEG method

**Fig. 1:**
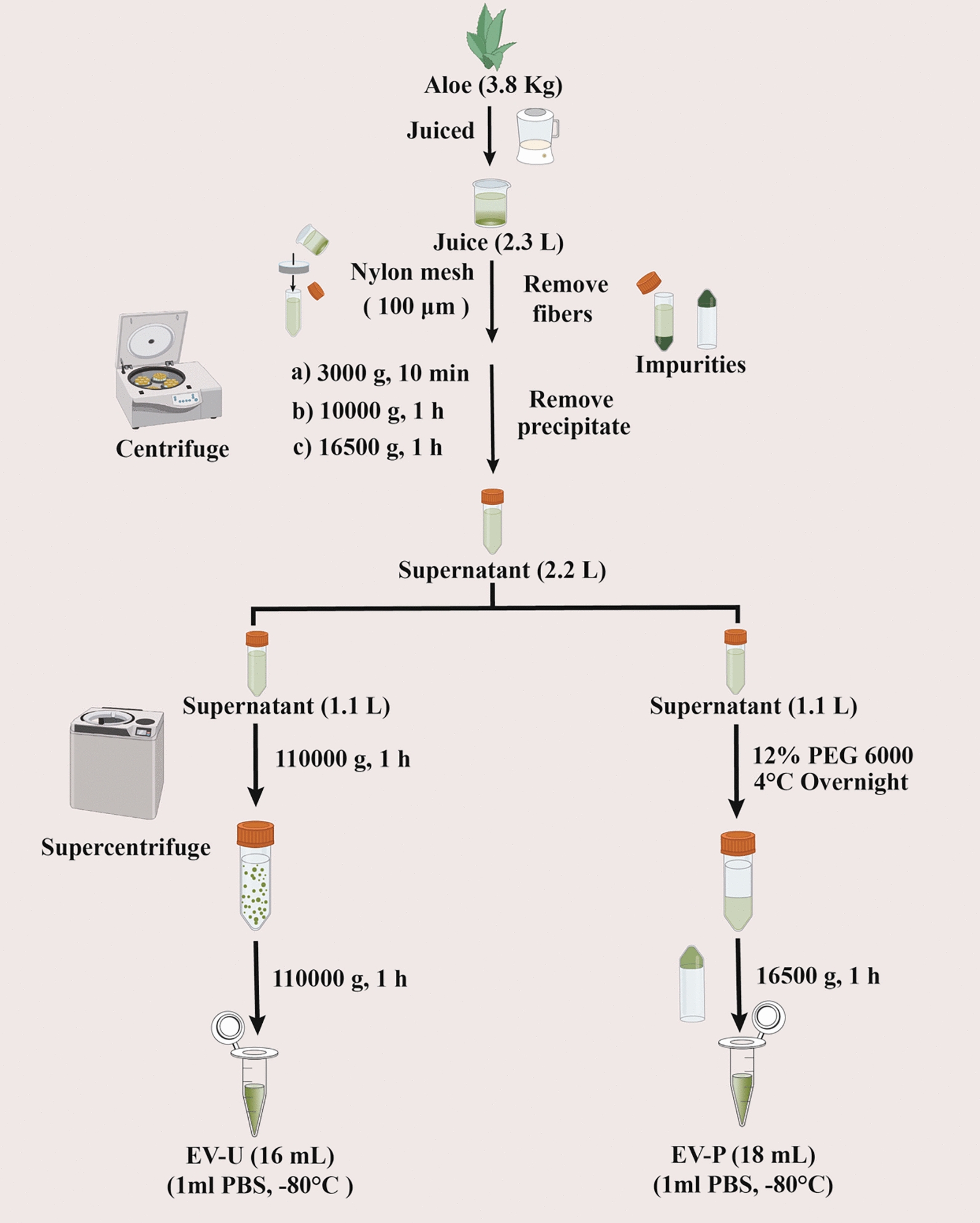
Isolation and purification of AV-EVLPs by differential ultra-centrifugation and polymer precipitation with PEG-6000, respectively

3.8 kg of fresh leaves were chopped into small pieces, juiced and filtered through a nylon mesh (100 µm) to remove excess fibers. The resulting juice underwent sequential differential centrifugation at 3000×*g* (10 min), 10,000×*g* (60 min) and 16,500×*g* (60 min) to remove large fiber cells, which afforded 2.3 L of supernatant. To compare the two methods in parallel, 2.2 L of the supernatant was divided equally into two parts (1.1 L each) for the dual purification of AV-EVLPs (Fig. 1). EV-U was isolated via ultra-centrifugation (110,000×*g*, 60 min) twice, while EV-P was precipitated with PEG-6000 at a final concentration of 12% (w/v) overnight at 4 °C with gentle shaking followed by centrifugation (16,500×*g*, 60 min) according to the method described by Kalarikkal et al. [28]. Finally, the protein concentration of the samples EV-U and EV-P was determined using a BCA assay kit.

### Characteristics of AV-EVLPs

10 μL of the sample was applied to a carbon-coated copper grid, negatively stained with 10 μL of 1% phosphotungstic acid for 2.5 min and air dried successively. Finally, their surface morphology was observed by transmission electron microscopy (TEM, HITACHI, HT-7800, Tokyo, Japan).

The size distribution was measured by nanoparticle tracking analysis (NTA) using a Nanosight NS300 system (Malvern, U.K.) equipped with a blue laser (488 nm) and software version 3.3 (Malvern, U.K.). The EVs isolated were diluted with PBS to achieve a concentration between 10^7^ and 10^9^ particles/mL. The sample was injected into a sample chamber and measured in triplicate with a capture time of 30 s.

### Composition analysis of AV-EVLPs

Biphasic liquid–liquid extraction protocol with methyl *tert*-butyl ether/methanol and an SCIE series HPLC interfaced to an Applied Biosystems 5500 triple quadrupole mass spectrometer was used to extract and monitor the lipid compositions of AV-EVLPs according to a previously reported method.

The protein composition differences between AV-EVLPs obtained by two methods were compared by electrophoresis performed on a 12% PAGE gel and stained with Coomassie blue for 1 h after extraction with RIPA lysis buffer (1:5, *v*/*v*).

The chemical profile of fresh Aloe, EV-U and EV-P extracts were determined using UHPLC/QTOF-MS after ultrasound extraction with 80% methanol. The condition of chromatographic separation and mass detection was almost the same as the method mentioned previously [29, 30]. Water containing 0.1% formic acid (solvent system A) and acetonitrile (solvent system B) served as the mobile phase. The only difference is the change of elution gradient, which was listed as follows: 0–7 min, 10–40% B; 7–8 min, 40–99% B; 8–10 min, 99–10% B.

### Cell culture

The pancreatic cancer cell line (Panc-1) and normal pancreatic cells (H6c7) were cultured in DMEM medium supplemented with 10% fetal bovine serum (FBS), penicillin (100 units/mL), and streptomycin (100 μg/mL) at 37˚C with 5% CO_2_ and saturated humidity, and the culture medium was replaced every other day.

### Cell uptake

To demonstrate the uptake of EV-U and EV-P by Panc-1, they were stained with DIO green dye at 37 ˚C for 30 min and then filtered by ultrafiltration (100 kDa) to remove free dye. Subsequently, Panc-1 cells were cultured in DIO-labelled EVLPs medium and separately fixed 1, 3 and 5 h. Finally, the cells were examined using Olympus IX73 fluorescence microscopy after washed with PBS and nuclear stained with the fluorescent dye DAPI for 5 min.

### In vitro antitumor activity

#### MTT assay for cell viability

Panc-1 and H6c7 with a density of 8 × 10^3^ cells/well were seeded in 96-well plates. After treatment with series gradient concentrations (25, 50, 100, 200, 400, 800, 1000 μg/mL) of AV-EVLPs for 24 h, a final concentration of MTT (0.5 mg/mL) was added to cells and the plate was then incubated for additional 4 h until the reaction was terminated by DMSO. Finally, the absorbance at 570 nm was measured using a spectrophotometer (BioTek Instrumentals, Inc., Winooski, VT, USA).

#### Wound healing and Transwell assay for cell migration and invasion

Cells were seeded in 6-well plates and incubated until they reached approximately 90% confluence. The cell migration was evaluated by wound region imaged under microscope after two parallel lines were formed using a 10 μL pipette tip and intervention with or without AV-EVLPs at 0 and 24 h, respectively.

Cellular invasion was assessed using matrigel-coated transwell inserts. Briefly, cells treated with EVs were suspended in DMEM and evenly spread in the upper chamber. The lower chamber was filled with 800 μL of DMEM containing 20% FBS. After 24 h of incubation, the cells were fixed with 4% paraformaldehyde and stained with crystal violet. The number of cells was then counted by photographing under an Olympus BX53 microscope.

### In vitro verification of cell pyroptosis

#### Intracellular ROS assay

ROS levels were measured using an assay kit with DCFH-DA as probes according to the manufacturer’s instructions. After EV-U and EV-P treatment, Panc-1 cells were incubated with serum-free medium containing DCFH-DA (10 μM) at 37 °C for 20 min. The fluorescence images were observed and recorded using a fluorescence microscope.

#### Tunnel assay

Pyroptosis was detected using one step TUNEL apoptosis assay kit according to the manufacturer’s protocol. Briefly, cells were fixed, permeabilized and incubated with TdT enzyme and fluorescein-labeled dUTP to specifically label 3′-OH DNA termini. Nuclei were counterstained with DAPI (0.5 μg/mL) to visualize nuclear morphology. After capturing the images using a fluorescence microscope, signal intensity was quantified using ImageJ software. TUNEL-positive cells were identified by distinct red fluorescent signals colocalized with DAPI-stained nuclei, while negative controls showed no specific fluorescence.

#### TEM observe for cell morphology

After treatment with 250 μg/mL of extracted AV-EVLPs for 24 h, Panc-1 cells were collected and fixed with 5% malondialdehyde and 1% osmic acid after intermittent rinse with PBS. Subsequently, a series of sections are examined using a TEM for topography examination following the application of routine staining, soaking and fixation with epoxy resin.

#### ELISA for IL-1β, IL-18 and LDH levels

The level of IL-1β and IL-18 were measured using an ELISA kit according to their respective optical detection absorbance at 450 nm. Pyroptosis was further evaluated by detecting the level of LDH released into culture supernatants using the assay kit.

### In vivo antitumor activity

Twenty female BALB/c-nude mice (15–16 g body weight, approximately 5–6 weeks old) were purchased from the animal center of Changsheng Bio-technology Co., Ltd. They were housed in ambient conditions (22 ± 2 °C) with a 12-h light/12-h dark cycle.

According to the review summed by Chu et al. [31], 1.5–6 mg/kg or 100–250 μg/mice of p-EVLP are often intraperitoneally or intravenous administered to xenograft tumor mice model for 2–3 weeks when evaluate their anti-tumor activities. Accordingly, the dosage of 2 and 4 mg/kg and the administration periods of every other day for 2 weeks were optimized through preliminary experiment evaluation. After 7 days of adaptive feeding, each mouse received a subcutaneous injection of 1 × 10^7^ Panc-1 cells in the right axilla. One week after the tumor-bearing process, the mice were randomly divided into four groups (*n* = 5), namely control (saline), EV-U (2 and 4 mg/kg) and gemcitabine (5 mg/kg) group. The body weight as well as tumor volume calculated by length and width according to the formula (V = L × W^2^/2) were recorded and calculated every other day for 3 weeks, and 100 μL of drug solution were simultaneously administered by intraperitoneal injection.

12 h after the final administration, tumor tissues were harvested and divided into two parts, one was fixed in 4% paraformaldehyde for histological observation and immunohistochemically stained for Ki67, IL-1β and IL-18, the other was immediately stored in −80 °C for the subsequent molecular biology validation experiments. Other tissues (heart, liver, spleen, lung, kidney and pancreas) were collected and fixed in 4% paraformaldehyde solution for further drug safety evaluation analysis.

All experimental protocol was approved by the Ethics Review Committee for Animal Experimentation of Dalian Medical University (approval number: AEE23083).

#### Western blot and quantitative real-time PCR (qRT-PCR) for proptosis-related mechanism validation

The expression of proliferation and proptosis-related molecules was evaluated using Western blot and qRT-PCR. The total protein of panc-1 cells and tumor tissues was extracted and concentration quantified by the RIPA reagents and BCA method, respectively. Equal amount of protein was electrophoretically separated by 10% SDS-PAGE, transferred using a PVDF membrane and then blocked in 5% milk with TBST for 2 h. Primary antibodies were added overnight at 4 °C followed by incubation with horseradish peroxidase (HRP) conjugated secondary antibodies for 2 h at room temperature. Bands were detected using ECL Plus chemiluminescence reagent and quantified using Image J software (National Institutes of Health, USA).

Similarly, the RNA of Panc-1 cell and tumor tissue was respectively extracted, and reverse transcribed to cDNA using Trizol reagent and reverse transcription kit. Subsequently, the samples were amplified by SYBR green qPCR Kit using primers (Supplementary Table S1) and detected by BIOER 9600 qRT-PCR system. All samples were quantitated using the comparative CT method by normalization to the amount of β-actin.

#### Statistical analysis

All values were expressed as mean ± standard deviation (SD). Differences between different groups were analyzed and compared with one-way analysis of variance (ANOVA) using GraphPad Prism 9.5.0. The value *p* < 0.05 was considered as statistically significant.

## Result

### Characterization and composition analysis of EV-U and EV-P

The yield and quality of the AV-EVLPs obtained according to the isolation steps described in Fig. 1 was quantitatively measured and compared. After a series of processing steps, 16 and 18 mL of the EV-U and EV-P samples was obtained, respectively. This slight difference in recovery efficiency may arise from variations in feature extraction principles between the two methods. Specifically, ultracentrifugation directly pellets AV-EVLP under high gravitational force, whereas PEG precipitation relies on intermolecular interactions to enrich particles, potentially retaining more liquid volume but possibly introducing soluble impurities (e.g., free proteins or polymer residues).

**Fig. 2:**
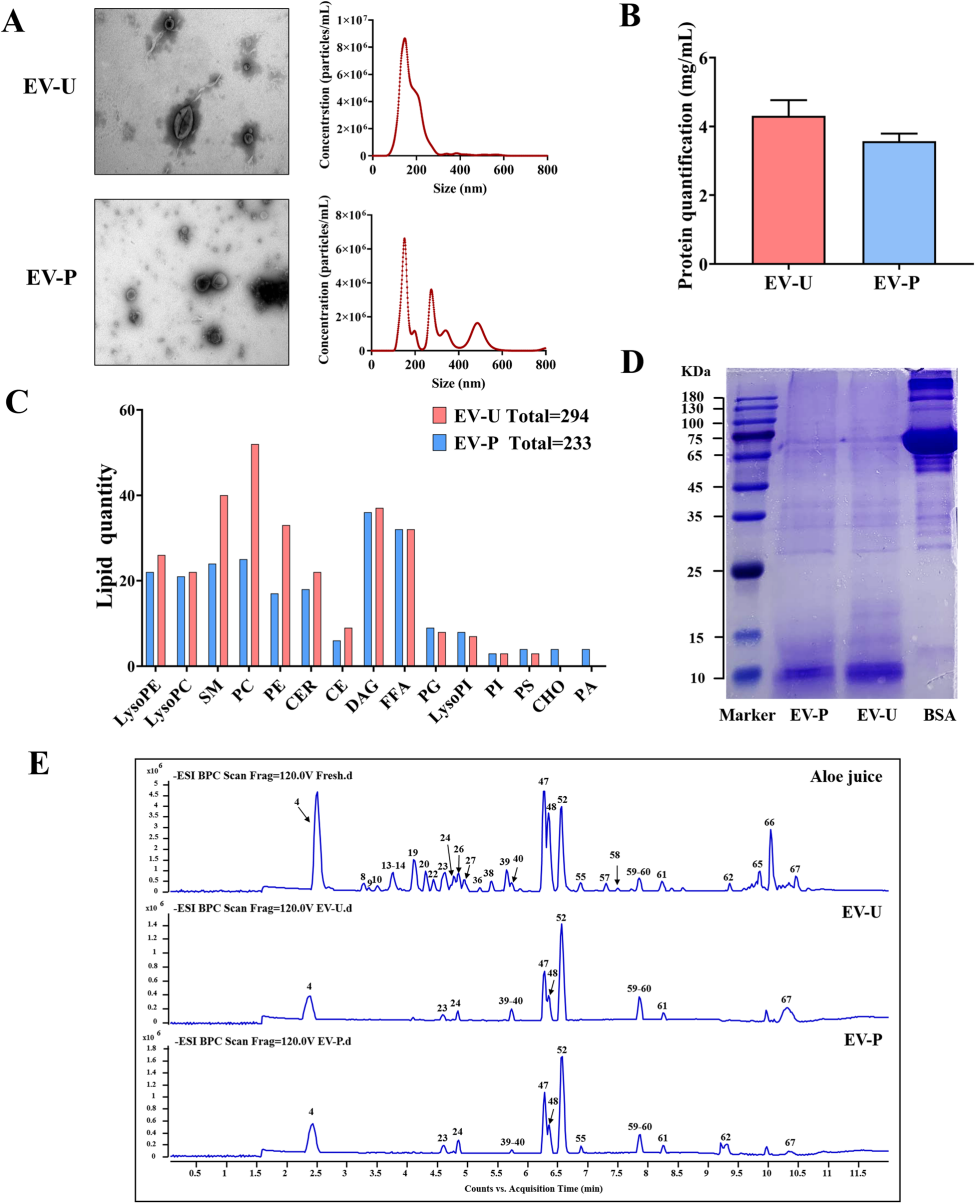
Characterization of Aloe vera-derived extracellular vesicle-like particles. **A** Morphological and size characterization of AV-EVLPs performed using TEM and NTA. Scale bar = 200 nm. **B** Protein concentration of AV-EVLPs quantitatively measured by bicinchoninic acid (BCA) assay. **C** Lipid species identified in AV-EVLPs using histograms. Further details can be found in Supplementary Table S2. **D** The protein of AV-EVLPs separated by 12% Bis–Tris protein gel and visualized with Coomassie blue. **E** Representative base peak chromatogram of fresh Aloe juice, EV-U and EV-P in negative ion mode. Further details can be found in Supplementary Table S3

Despite EV-U group had a slightly lower volume, its particle size (7.87 × 10^8^ ± 2.08 × 10^7^ particles/mL) obtained by NTA assay was higher than that of EV-P (4.65 × 10^8^ ± 8.17 × 10^7^ particles/mL) (Fig. 2A). Similarly, the BCA assay results indicated a significantly higher protein concentration of EV-U (4.31 ± 0.46 mg/mL) than that obtained with EV-P (3.57 ± 0.22 mg/mL) (Fig. 2B). Besides, Plant-derived EVs are exosome-like nanovesicles characterized by a phospholipid bilayer structure and saucer-shaped or cup-shaped morphology, with diameters ranging between 40 and 500 nm. As expected, both EV-U and EV-P exhibited distinct ‘cup-shaped’ double-membrane structures under TEM, with mean diameters of 179.3 nm and 227.1 nm, respectively (Fig. 2A). Moreover, EV-U demonstrated fewer impurities and more uniform dispersion, as evidenced by the narrower size distribution.

Lipid composition analysis showed that 294 and 233 kinds of lipid has been identified in EV-U and EV-P, respectively. Among them, phospholipid (LysoPE, LysoPC, PC), sphingolipids (SM), glycerolipids (DAG) and fatty acyls (FFA) are the main enriched categories (Fig. 2B). Additionally, richer PC and PE observed in EV-U and specific cholesterol (CHO) and PA displayed in EV-P highlights compositional differences between the two extracted method (Supplementary Table S2).

As an important constitution of AV-EVLPs, similar protein band were displayed by SDS-PAGE. Specifically, a distinct band within the 15–25 kDa range was uniquely observed in EV-U (Fig. 2C). Similarly, chemical composition analysis identified 45 and 44 constituents in EV-U and EV-P, which closely matched the phytochemical profile of their homologous fresh Aloe vera (Fig. 2D; Supplementary Table S3).

### Cell uptake

**Fig. 3:**
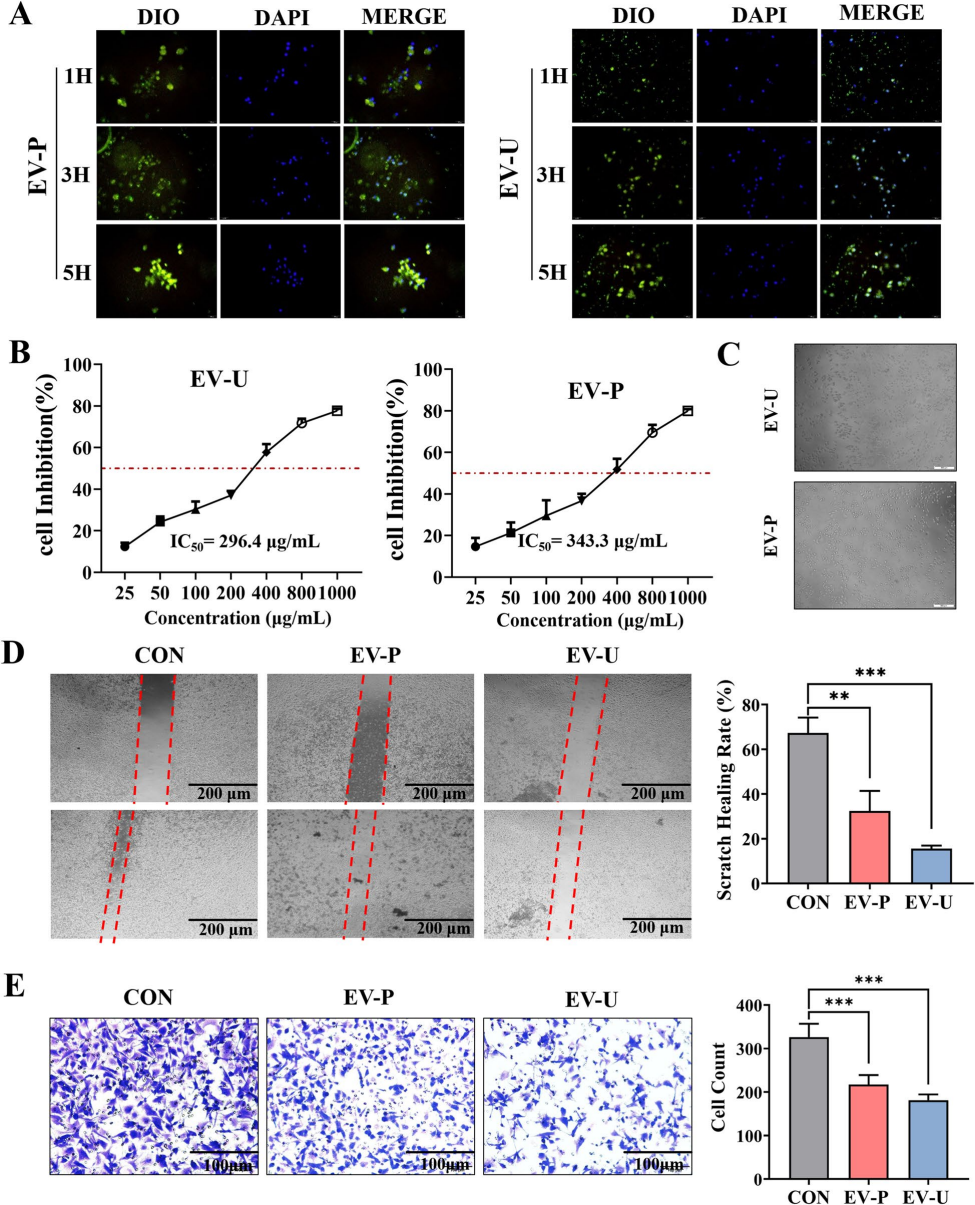
Cell uptake and inhibition of AV-EVLPs on the migration and invasion of Panc-1. **A** The uptake of AV-EVLPs after 1, 3 and 5 h fix was observed using fluorescence microscopy. The nuclear and EVs were stained and labelled with DAPI and DIO, respectively. Scale bar = 100 μm. **B** The cell viability of Panc-1 treated with different concentrations of AV-EVLPs for 24 h were evaluated using MTT assay. **C** Morphological features of Panc-1 after treatment with AV-EVLPs at IC_50_ for 24 h. **D**,**E** Cell migration and invasion was detected by wound healing and Transwell assay. * *p* < 0.05, ** *p* < 0.01, *** *p* < 0.001

The intracellular bioactivity of extracellular vehicles critically relies on their efficient cellular internalization. Accordingly, an uptake kinetic study using DiO-labeled EVLPs co-incubated with Panc-1 cells for 1, 3, and 5 h was conducted to systematically evaluate the time-dependent cellular internalization. As shown in Fig. 3A, a growing tendency towards confluent has been obviously observed over time under fluorescence microscopy, which displayed peripheral localization at 1 h (early endocytosis), diffuse cytoplasmic distribution at 3 h (vesicular trafficking), and enhanced membrane fusion at 5 h (Supplemental Fig. S3). Notably, higher fluorescence intensity displayed by EV-U than EV-P at 5 h reflecting its superior uptake efficiency. Our data not only validates that AV-EVLPs can be taken up by Panc-1 cells but also establishes a mechanistic basis for their subsequent enhanced therapeutic efficacy observed in vivo.

### AV-EVLPs inhibited migration and invasion behavior of Panc-1 cells

In order to evaluate the efficacy and specificity, the inhibitory effects of extracted AV-EVLPs on the proliferation of Panc-1 and H6c7 were first detected using MTT assay. As shown in Fig. 3B, both EV-U and EV-P decreased the cell viability of Panc-1 in a dose dependent manner, with respective IC_50_ value of 296.4 and 373.3 μg/mL. Additionally, the appearance of cytoplasmic swelling and membrane rupture was also observed under light microscopy (Fig. 3C). Crucially, neither EV-U or EV-P exhibited toxicity towards normal H6c7 cells (Supplemental Fig. S1). Subsequent migration and invasion assays using two kinds of AV-EVLPs with screened IC_50_ dosage demonstrated significant suppression of Panc-1 compared to untreated controls (*p* < 0.01, Fig. 3D, E).

### AV-EVLPs induced the release of proinflammatory factors (ROS, LDH, IL-1β, IL-18)

**Fig. 4:**
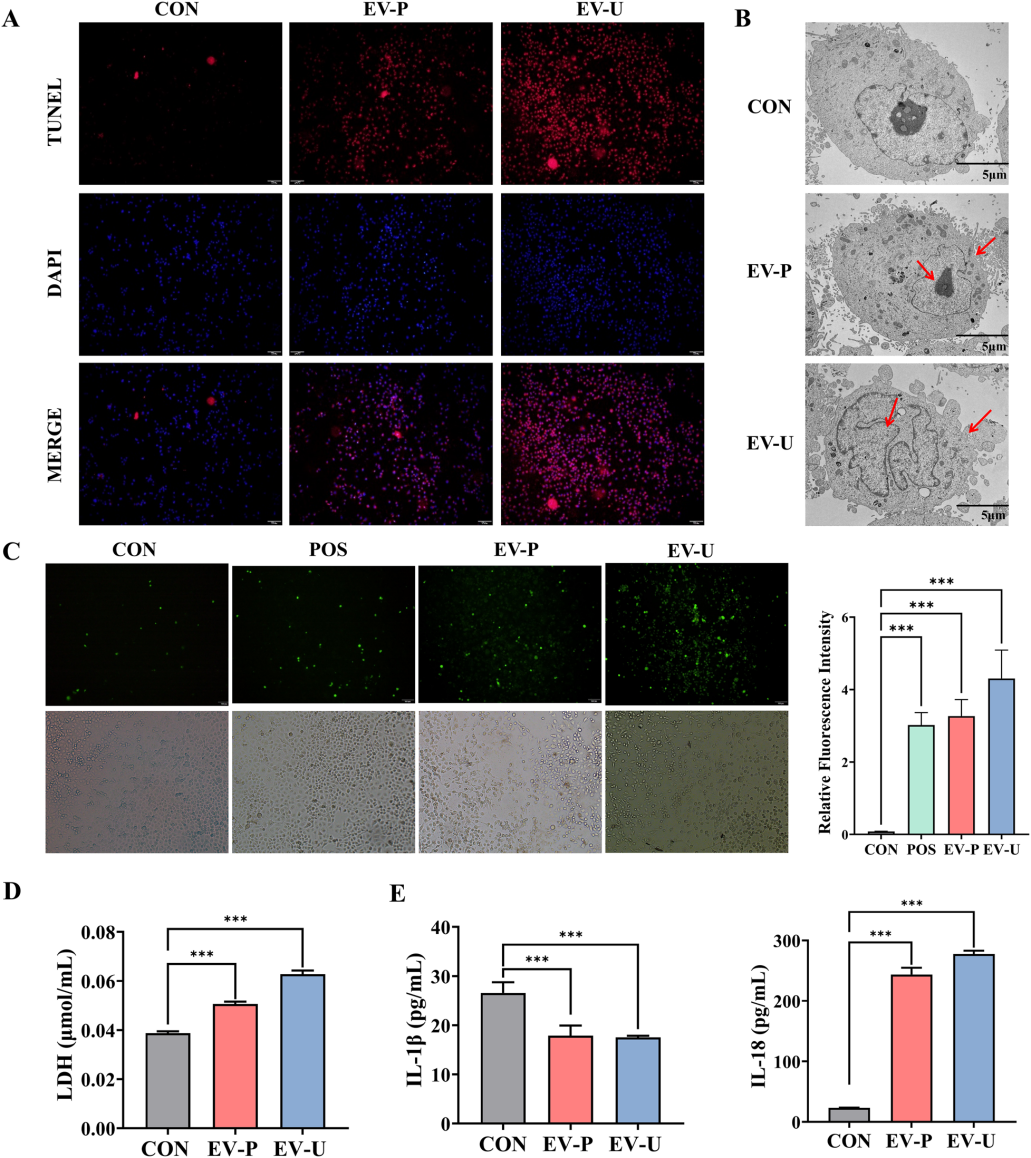
AV-EVLPs induce pyroptosis in Panc-1 cells. **A** TUNEL staining assay of Panc-1 after AV-EVLPs treatment, scale bar = 100 μm. The data analysis of mean fluorescence intensity in vitro could see in Fig. S4. **B** Pyroptosis of Panc-1 after AV-EVLPs treatment detected by TEM. Red arrows point to prosomes formed in cells and ruptured cell membranes. Scale bar = 50 μm. **C** Fluorescence images of the intracellular ROS levels using DCFH-DA, scale bar = 100 μm. **D** The LDH activity in culture mediums after AV-EVLPs treatment. **E** The levels of IL-1β and IL-18 in Panc-1 was detected by ELISA. The data represent as mean ± SEM from three biological replicates. *** *p* < 0.001

Pyroptosis, a crucial process for tumor immune surveillance, manifested by DNA fragmentation, cytoplasmic swelling, membrane rupture, inflammasome activation and pro-inflammatory cytokines release. TUNEL analysis revealed significant DNA fragmentation in EVLPs-treated cells, characterized by punctate or diffuse red fluorescence signals within nuclei (Fig. 4A). Further quantitative analysis demonstrated that the mean fluorescence intensity of TUNEL-positive cells in the EV-U group was respective 3- and 1.8-fold higher than in the control and EV-P group (Supplemental Fig. S3), which was consistent with their pro-pyroptotic activity. Besides, DAPI staining confirmed nuclear integrity, excluding nonspecific membrane damage.

Morphology characteristics of chromatin consolidation, cytosolic vacuolation and membrane vesiculation was clear observed in the EV-U group (Fig. 4B). ROS has been demonstrated to be a key factor in inflammasome activation. It can directly target inflammasome-associated caspase proteins, lead to the cleavage of pro-IL1β and pro-IL18 before their secretion and mediate the pore-forming activity of GSDMD/GSDME [32]. As expected, DCFH-DA staining confirmed elevated ROS levels in AV-EVLPs treated cells, with EV-U showing approximately 20% higher fluorescence intensity than EV-P (Fig. 4C). Besides, we also observed that the intensity of ROS fluorescence was highest in the ruptured cell at the moment of bursting (Supplemental Fig. S2).

Furthermore, the levels of IL-1β, IL-18 and LDH further evidenced the pyroptosis after EVs treatment. As shown in Fig. 4E, the IL-1β level of control group (26.60 ± 2.16 pg/mL) was higher than that of EV-U (17.51 ± 0.33 pg/mL, *p* < 0.001) and EV-P (17.86 ± 2.05 pg/mL, *p* < 0.001) group. In contrast, the IL-18 level in the control group (23.21 ± 0.42 pg/mL) was significantly lower than that of EV-U (277.54 ± 5.47 pg/mL) and EV-P (243.71 ± 11.20 pg/mL) group (*p* < 0.001, Fig. 4E). Like IL-18, the LDH released was also increased by the AV-EVLPs (Fig. 4D). Collectively, the AV-EVLPs prepared by ultra-centrifugation demonstrated superior bioactivity compared to PEG-based preparations. Accordingly, EV-U was chosen for subsequent in vivo validation.

### Aloe-EVs promoted GSDMD/E-mediated pyroptosis by activating the caspase-1,3,7,9 signaling pathway in Panc-1

**Fig. 5:**
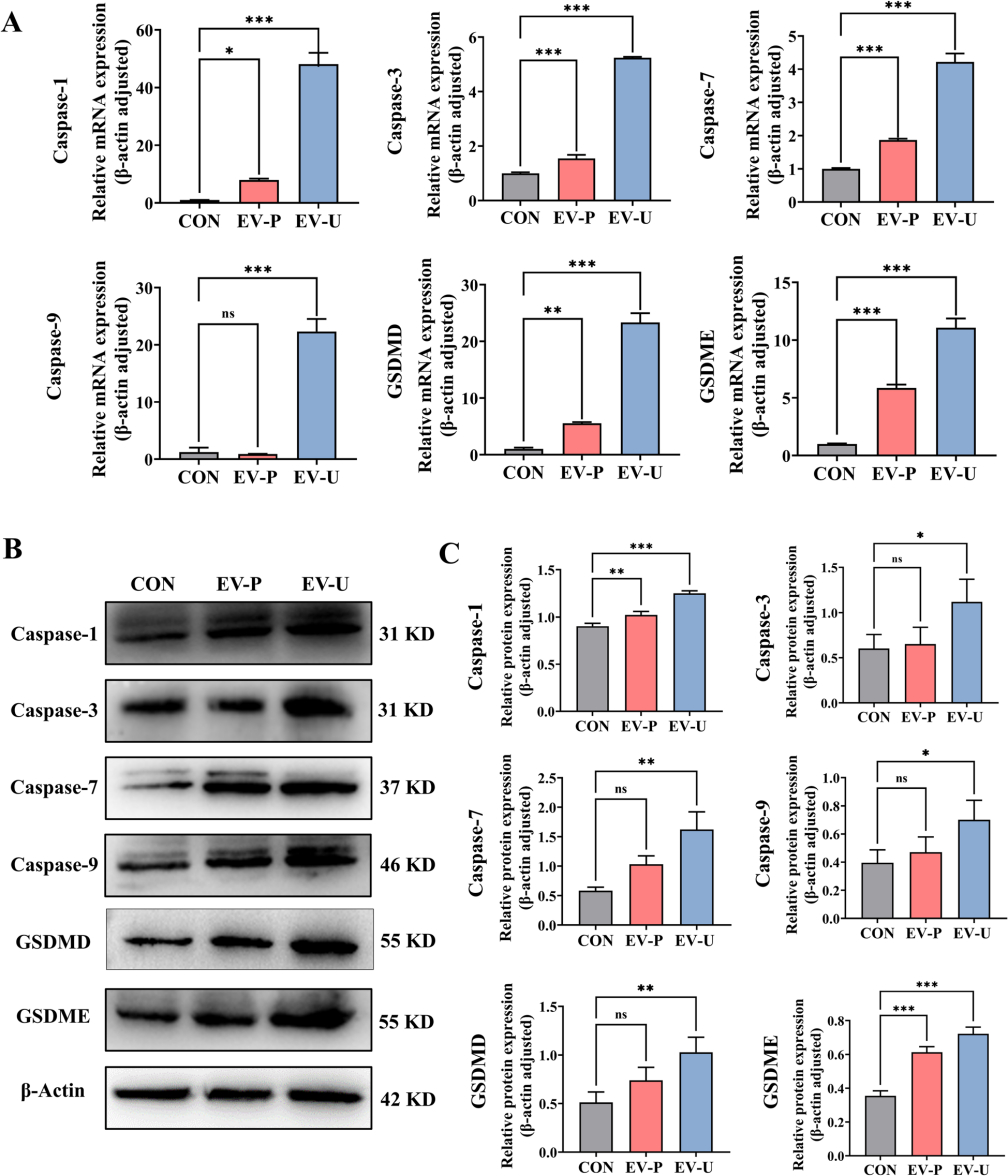
AV-EVLPs promoted GSDMD/E-mediated pyroptosis by activating the caspase-1,3,7,9 signaling pathway in Panc-1. **A**,**B** Real-time quantitative reverse transcription and Western blotting to analysis the genes and protein expression of caspase-1,3,7,9 and GSDMD/E in Panc-1. **C** Statistical analysis of protein expression level. The data represent as mean ± SEM from three biological replicates. * *p* < 0.05, ** *p* < 0.01, *** *p* < 0.001

To delineate the molecular mechanisms underlying AV-EVLPs induced pyroptosis, we further investigated the expression levels of caspase family that participate into the classical caspase-1/7-GSDMD and non-classical caspase-3/9-GSDME dependent pyroptosis pathways [33]. As expected, both proteins and RNA expression of caspase-1/3/7/9 and GSDMD/E were increased by the AV-EVLPs treatment, with EV-U showing superior efficacy compared to EV-P (Fig. 5).

### EV-U inhibited pancreatic tumor growth in xenograft models

**Fig. 6:**
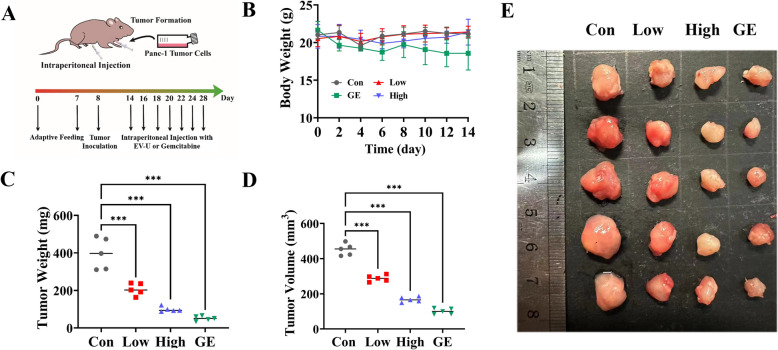
EV-U treatment inhibited tumor growth in mice with xenograft tumors. **A** The schedule of EV-U treated mice with xenograft tumors. **B**–**D** Effect of EU-V (2 or 4 mg/kg) and gemcitabine (5 mg/kg) on body weight, tumor weight and volume (*n* = 5). **E** The photography images of the tumor tissues

The effect of EV-U on tumor growth in vivo was further performed using xenograft tumor model subcutaneously transplanted with Panc-1 cell (Fig. 6A). As for the body weight, a significant decrease observed in the gemcitabine-treated group could be attributed to the side effects by chemotherapy, whereas EV-U administration maintained normal growth parameters (Fig. 6B). At the end of the experiment, EV-U could reduce tumor volume and weight compared with the control and the efficacy of high-dose group is equivalent with that of gemcitabine (Fig. 6C–E).

**Fig. 7:**
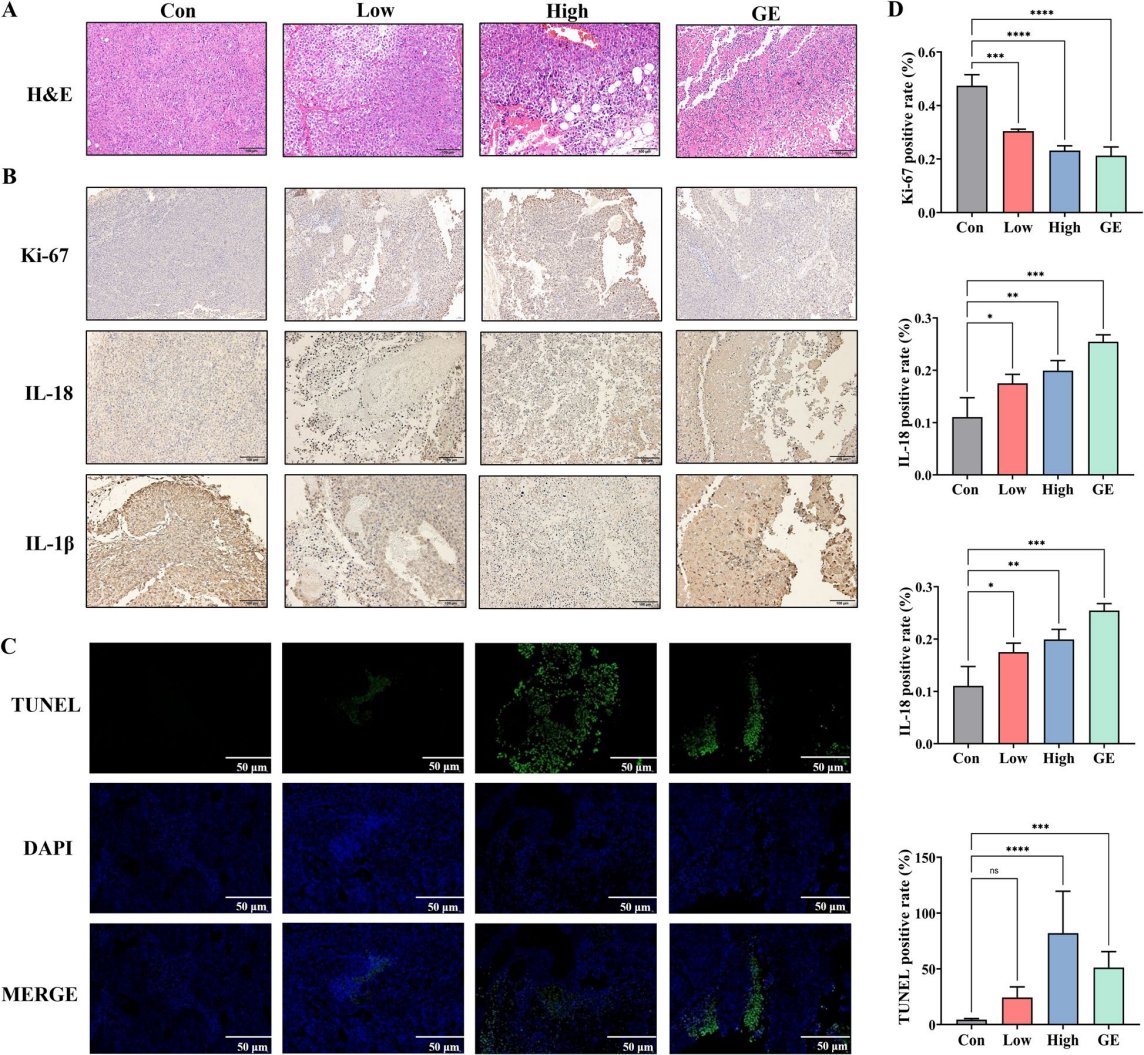
H&E staining (**A**), immunohistochemistry for Ki-67, IL-18 and IL-1β (**B**), TUNEL staining (**C**) and their positive rate (**D**) on tumor tissues from animal models. Data from three technical replicates are shown. Scale bar = 100 µm or 50 µm

Histopathological analysis revealed tumor architecture disintegration with vacuolization as well as reduced inflammatory infiltrates and nuclear atypia in EV-U and gemcitabine group, particularly in the high-dose group (Fig. 7A). No abnormalities in major organs (heart, liver, spleen, lung and kidney) further supports the safety evidence of EV-U without toxicity and immunogenicity (Supplemental Fig. S5). Immunohistochemical analysis demonstrated decreased Ki-67 positivity following treatment, suggesting that EV-U might potentiate the chemosensitivity of tumor tissues, thereby exerting marked anti-proliferative effects (Fig. 7B). Concurrently, TUNEL detection revealed that the intensity of green fluorescence labelled in the tumor tissue of high dose EV-U group was significantly greater than that of the other groups (Fig. 7C). These findings supported the evidence that EV-U is a promising candidate for pancreatic carcinoma therapy.

Besides, the expression of IL-1β and IL-18 in tumor tissues was also evaluated by immunohistochemical analysis (Fig. 7B). When compared to the control group, EV-U group with both doses exhibited markedly reduced IL-1β and elevated IL-18 expression (Fig. 7D), respectively. The change tendency was aligning with the cellular data as mentioned earlier (Fig. 4D). Notably, unlike EV-U, increased IL-1β expression by gemcitabine is consistent with prior reports that chemotherapy agents activate the NLRP3 inflammasome to promote IL-1β secretion. Moreover, upregulated IL-18 levels by both EV-U and gemcitabine are considered relevant to enhanced caspase-1 activity and GSDMD/E cleavage, which supports the role as a pyroptosis effector synergizing with cytotoxic T-cell responses to suppress tumors. This dual modulation of IL-1β suppression and IL-18 activation highlights unique advantage of plant-derived EVs in targeting cancer-associated inflammation.

### EV-U promoted GSDMD/E-mediated pyroptosis by activating the caspase-1,3,7,9 signaling pathway in vivo

**Fig. 8:**
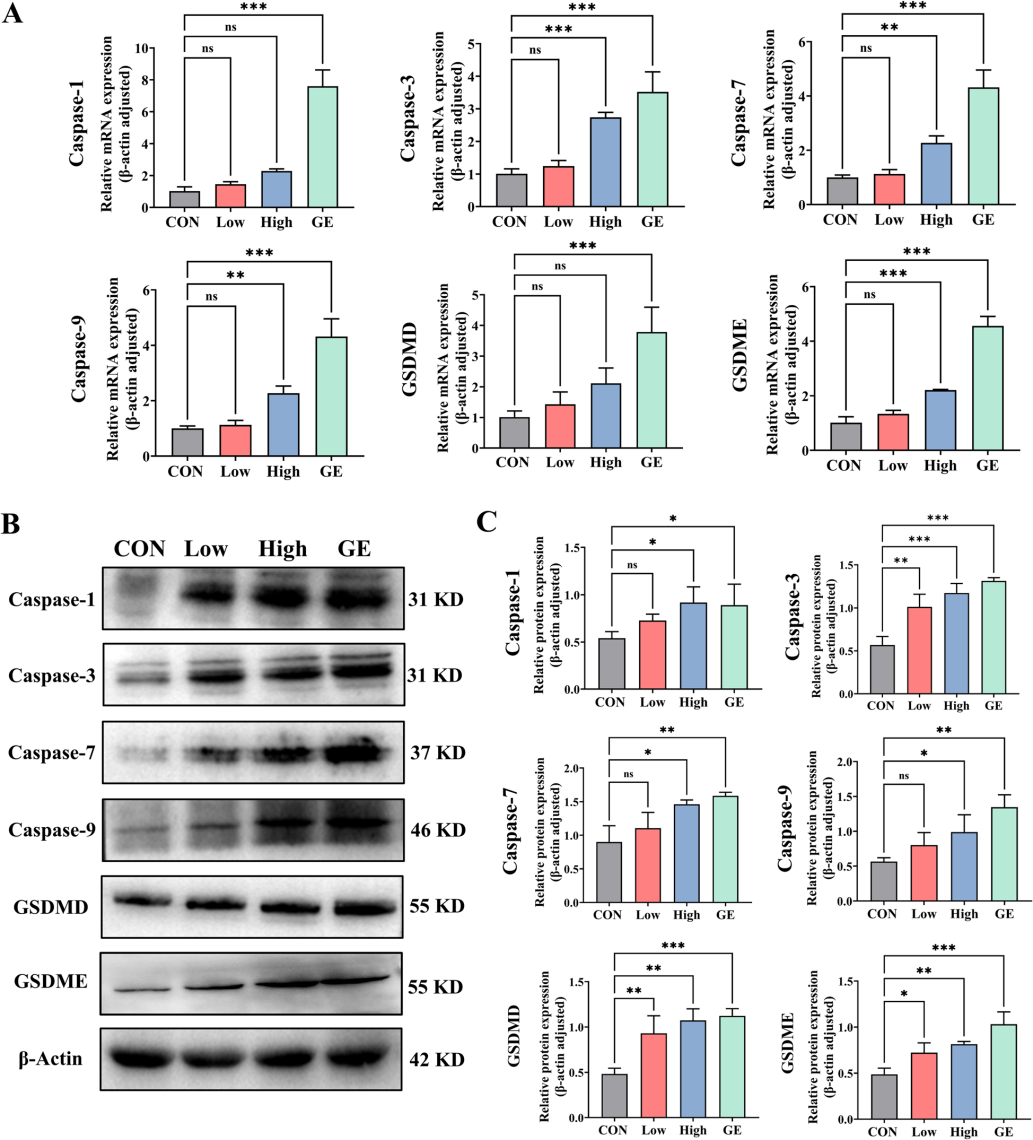
EV-U promoted GSDMD/E-mediated pyroptosis by activating the caspase-1,3,7,9 signaling pathway in vivo. **A** Real-time quantitative reverse transcription to analysis the expression of genes caspase-1,3,7,9 and GSDMD/E in xenograft tumors. **B** Western blotting method to identify the protein expression of caspase-1,3,7,9 and GSDMD/E in xenograft tumors. **C** Statistical analysis of protein expression level. The data represent as mean ± SEM from three biological replicates. * *p* < 0.05, ** *p* < 0.01, *** *p* < 0.001

To confirm that EV-U exerted anti-tumor effects through cell pyroptosis, the proteins and genes expression of the same targets as that of the cell in tumor tissue were also evaluated. And similar tendency was obtained at the animal level. Notably, GSDME is typically down-regulated in pancreatic tumor tissues due to promoter hypermethylation in others work, while EV-U could increase the GSDME expression and further enhance the cellular pyroptosis response (Fig. 8).

## Discussion

In recent years, p-EVLP have emerged as a research hotspot in view of their biomarker potential for early screening, diagnosis and prognosis [34]. People have gradually recognized the therapeutic significance of EVLPs as they carry specific combinations of proteins, nucleic acids, metabolites and lipids. However, clinical translation of serum or body fluid-derived EVLPs poses challenges in terms of the component traceability and biochemical heterogeneity between different batches [35]. p-EVLPs present distinct advantages over mammalian counterparts, offering scalable source, safety, high biocompatibility and low immunogenicity, has demonstrated the potential of drug delivery and physiological barrier crossing in the tumor field (breast, colon, liver cancer) [36, 37]. For example, ginger-derived EVLPs can not only regulate the host physiological environment as well as the composition and distribution of intestinal microorganisms through their own RNA, but also exhibit anti-inflammatory activity through encapsulated gingerol and shogaol [38]. In addition, Zhang et al. [39] demonstrated that the better anti-colon cancer activity of ginger-derived EVLPs compared to traditional liposomes is attributed to their higher loading capacity and intestinal aggregation of fatty acids on the surface.

It is generally accepted that pursuing high concentrations and purity of EVLPs from specimens is the primary and crucial step for further pharmacodynamic evaluation [40]. Currently, differential ultracentrifugation, polymer precipitation and immunomagnetic bead technology, which possess their own advantages and disadvantages, are the commonly adopted methods for p-EVLPs isolation. However, difference in physicochemical properties and functions among these techniques have not yet been systematically compared. Aloe vera, a typical tender and juicy plant that bears little relationship to seasonal cycles, has been brought into our sight. After optimizing a series of steps, EV-U and EV-P isolated by ultracentrifugation and PEG-6000 were characterized. Results showed that ultracentrifugation yields AV-EVLPs with more uniform particle sizes and fewer impurities, establishing it as the most reliable isolation method.

Nevertheless, this technology is restricted to specialized platforms due to substantial equipment costs and time-consuming procedures. Comparatively, although suitability for most laboratory operations, the AV-EVLPs isolated by polymer precipitation contain numerous impurities and less uniformity in particle size, which could impact the accuracy of the detection results. Moreover, the stability assessments revealed that the AV-EVLPs obtained by both styles remain stable when stored at −80 °C for 1, 15, 30, 180 and 360 days (Supplemental Fig. S6). Collectively, the PEG method could be adopted if you want to make a primitive study about efficacy and properties of p-EVLPs, while ultracentrifugation is recommended for further analysis and other evaluations. Accordingly, the anti-cancer activity of EV-U and EV-P was subsequently compared using Panc-1 cell.

From the perspective of pharmacological substance, several kinds of components such as anthraquinones, amino acids and polysaccharides contribute to the anti-tumor activity of Aloe vera by cell cycle arrest, apoptosis induction and inhibition of VEGF expression [41]. Obviously, EVLPs encapsulation enhances therapeutic performance by addressing conventional limitations of phytochemicals like poor water solubility and rapid clearance. Moreover, EV-U that possessed higher purity showed better biological activity than EV-P. Besides, the phenomenon of cell swelling and death was unexpectedly discovered during the course of our experiments, which is consistent with that of pyroptosis emerged in recent years [42]. Therefore, the pyroptosis pathway that is closely associated with suppressor of tumor progress has become the focus of follow-up mechanism research.

The active release of pro-inflammatory cytokines is a unique characteristic of pyroptosis compared to other forms of cell death. As the key indicator of pyroptosis, cytokines IL-1β and IL-18 formed by the cleavage and activation of caspase-1 exert their physiological functions after release through pores formed by Gasdermin D, whose levels is usually elevated [43]. In our research, the level of IL-18 is elevated in EVs treated group like most researchers, while that of IL-1β was slight reduced in the intervention group, which is contrary to recognized belief in the pyroptosis research. This inconsistency could be explained from the view of the relationship between IL-1β and pancreatic cancer, whose expression is increased in the carcinoma tissues compared to para-carcinoma tissue [44]. Typically, IL-1β has been identified as a driving factor which activates the stellated cells in TME, induces TAM-mediated immunosuppression and further promote the proliferation of connective tissue [45]. Besides, it has been confirmed that blocking IL-1β could enhance the anti-tumor activity of anti-PD-1 immunotherapy [46], underscoring its significance as a therapeutic target. Meanwhile, pretreatment with aloin (a typical component of Aloe) dose-dependently inhibited IL-1β-induced IL-8 production and IL-1β release in KB cells and LPS-induced Raw 264.7, respectively [47]. Accordingly, this dual immunomodulatory effect directly addresses the challenge of converting PC. Taken together, inducing pyroptosis is an effective approach to trigger a cascade of events in the cancer-immunity cycle, the steady-state switching mediated by the AV-EVLPs via silencing immunosuppressive IL-1β-driven pathways and amplifying IL-18-mediated antitumor immunity might reshape the TME from an immunologically “cold” into a “hot” phenotype, while this hypothesis remains to be verified through experiments in the follow-up study.

The in vivo uptake data confirm that EV-U can target tumor tissues through specific endocytotic mechanisms without causing toxicity to other healthy tissues (see Supplemental Fig. S7). Compared with the gemcitabine group, tumor growth was significantly inhibited in mice treated with EV-U, as characterized by decreased tumor volume and weight in the mice treated with the EV-U. The difference in body weight confirmed the safety of EVs. Additionally, H&E staining revealed that the control group exhibited deep nuclear staining, hypertrophy, abundant cytoplasm, occasional multinucleated cells, vigorous proliferation, tight arrangement, and clear intercellular boundaries. However, in the high-dose EV-U and gemcitabine groups, dispersed and ruptured cells, decreased density, leaked cytoplasm, enlarged vacuole structures as well as significantly condensed, fragmented and dissolved nuclei indicated cell necrosis. Ki-67 is a nuclear protein associated with cell cycle progression, which has been widely used as a proliferation marker for human tumor cells, reflecting the sensitivity of tumor cells to drugs [48]. The percentage of Ki-67 positive cells significantly increased after high-dose EV-U and gemcitabine intervention. This suggests that EVLPs can break the immune suppression of pancreatic tumor and make tumor cells sensitive to drugs. In summary, the results confirm that EV-U exerts antitumor effects through pyroptosis. This finding suggests that p-EVLPs are expected to be a nanotherapeutic agent that provides a potential strategy for tumor treatment.

Despite the research that focus on pyroptosis was blowout extending in the tumor field, previous studies have mostly linked the anti-tumor activity of p-EVLPs to mitochondrial apoptotic pathway activation and caspase-3 induction [49]. The idea introducing pyroptosis into the anti-cancer mechanism of AV-EVLPs originated from the accidental discovery of cell morphology with swollen and bursting after treatment (Supplemental Fig. S2), which have the typical feature of pyroptosis. Accordingly, in-depth indicators and signaling molecules participating into both classic and non-classic pathway were detected for the evidence of pyroptosis mechanism. In agreement with the empirical evidence, the activation of caspase-1 cleavage GSDMD was also confirmed in our research at the cellular and animal levels. Especially, GSDME serving as the downstream effector of activated caspase-3 was first identified in p-EVLPs. Caspase-3 is usually considered as the key protein in apoptosis in past cognition, whose promoter activity of pyroptosis formed by activation of GSDME was realized in the latest decade. Our study demonstrated that EVLPs inhibited tumor growth through caspases-3/9 activation and GSDME cleavage in Panc-1 cell and its xenograft models. Above research provides clues for unraveling the mystery of p-EVLPs against tumor.

Longer-term, encapsulating immunosuppressants within p-EVLPs demonstrates good market prospect, as evidenced by anti-colitis and anti-metastasis activities of methotrexate and miR-18a encapsulated p-EVLPs from ginger and grapefruit [50, 51]. However, their further explore has been constrained by the complexity of botanical ingredients and absence of clinically validated quality standards. Accordingly, future thought and exploration should prioritize developing specific solutions for these challenges.

## Conclusion

**Fig. 9:**
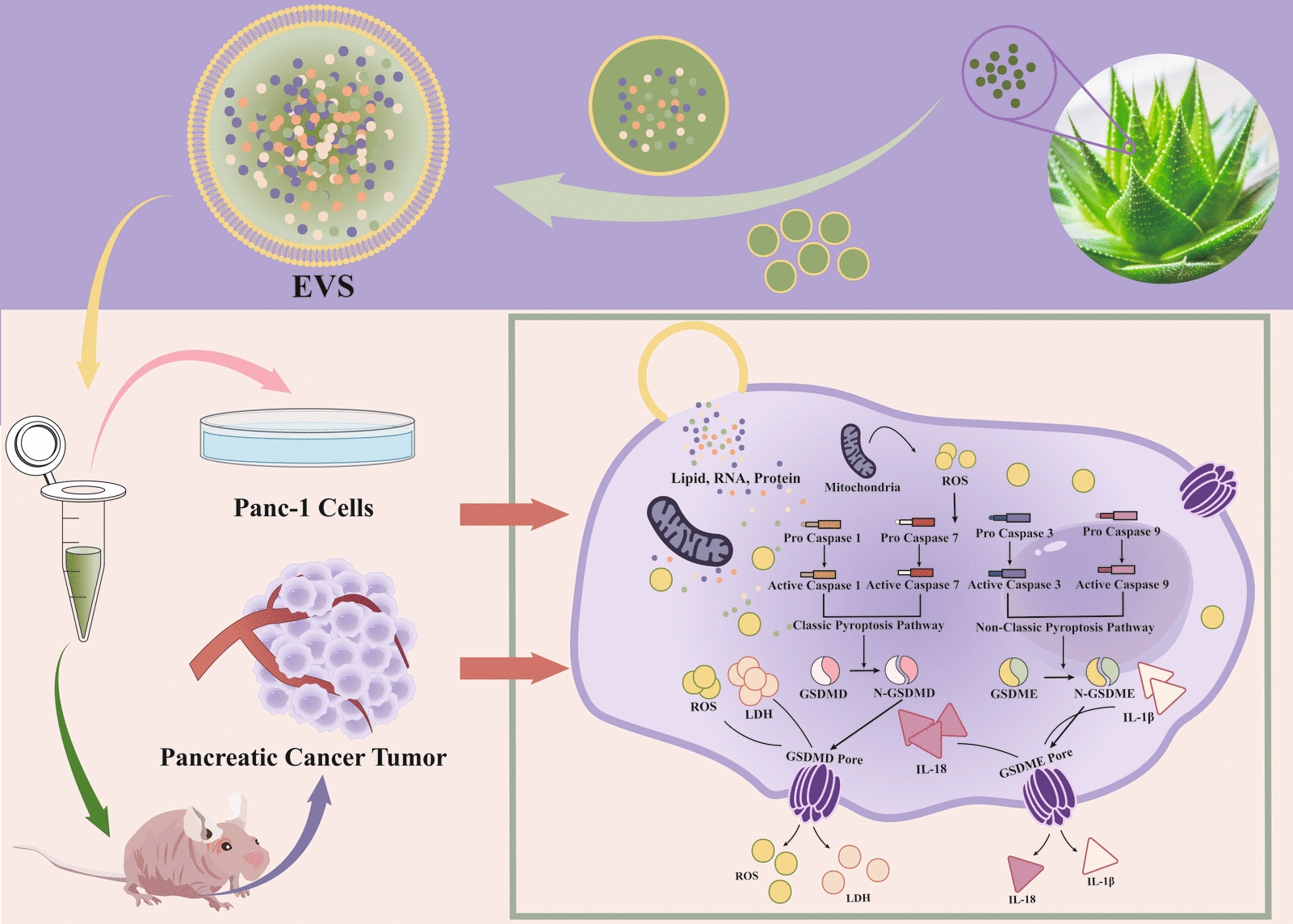
AV-EVLPs suppress pancreatic carcinoma progression through triggering pyroptosis via ROS-GSDMD/E pathways

This study demonstrates that EVLPs created from Aloe vera by ultra-centrifugation exhibit better activity than PEG-6000 due to their fewer impurities and more uniform dispersion. These AV-EVLPs can inhibit pancreatic cancer progression by increasing mitochondrial ROS release through the activation of caspase-1/3/7/9 and GSDMD/E-mediated pyroptosis in Panc-1 cells. Furthermore, EV-U demonstrated in vivo efficacy in a xenograft model, as evidenced by reduced tumor volume and weight without causing systemic toxicity or immunogenicity (Fig. 9). Therefore, this study not only enriches the understanding of pharmacodynamic material bases of Aloe Vera, but also provides a promising therapeutic strategy for PC from the insight of pioneering p-EVLPs as pyroptosis-inducing agents.

## Supplementary Information

Additional file 1.

## Data Availability

The datasets and all the relevant codes are available from the corresponding author.

## Abbreviations

AV-EVLPs: Aloe vera-derived extracellular vesicle-like particles
DIO: 3,3′-Dioctadecyloxacarbocyanine perchlorate
GSDMD: Gasdermin D
GSDME: Gasdermin E
IL-1β: Interleukin-1 beta
IL-18: Interleukin-18
NTA: Nanoparticle tracking analysis
PC: Pancreatic carcinoma
PDAC: Pancreatic ductal adenocarcinoma
PD-1: Programmed cell death protein 1
PD-L1: Programmed death-ligand 1
p-EVLPs: Plant-derived extracellular vesicles-like particles
TEM: Transmission electron microscopy
TME: Tumor microenvironment
TUNEL: Terminal-deoxynucleotidyl transferase mediated nick end labeling
UHPLC-MS: Ultra high-performance liquid chromatography-mass spectrometry
