# Supplementary material for: Aloe vera-derived extracellular vesicle-like particles suppress pancreatic carcinoma progression through triggering pyroptosis via ROS-GSDMD/E signaling pathway
Source: Chin Med. 2025 Jul 2;20:101. doi: 10.1186/s13020-025-01153-7 (PMC12219699; doi:10.1186/s13020-025-01153-7)
Supplement: Supplementary file 1 — Additional file 1. [file 13020_2025_1153_MOESM1_ESM.docx]

**Supplementary material**

**Aloe vera-Derived Extracellular Vesicle-Like Particles suppress pancreatic carcinoma** **progression through** **triggering pyroptosis via** **ROS-GSDMD/E signaling pathway**

Jieyu Shen ^a, b,^**^†^**, Tianfu Wei ^a,b,^**^†^**, Mingchen Li ^a,^**^†^**, Yuankuan Jiang ^a,b^, Jiahui Zhang ^a,b^, Yueyi Qi ^a,c^, Cai Chen ^a,b^, Xiaojie Li ^a,c^, Peng Huang ^a,b^, Jialin Qu ^a,*^

*^a^ Clinical Laboratory of Integrative Medicine, The First Affiliated Hospital of Dalian Medical University; No. 222, Zhongshan Road, Dalian 116011, China*

*^b^ Institute of Integrative Medicine, Dalian Medical University, No. 9, South Road of Lvshun, Dalian 116044, China*

*^c^ Institute (College) of Pharmacy, Dalian Medical University, No. 9, South Road of Lvshun, Dalian 116044, China*

**Legends for Figures and Tables**

**Fig.S1.** The cell viability of H6C7 treated with different concentrations of EV-P (**A**) and EV-U (**B**) for 24 h evaluated using MTT assay.

**Fig.S2.** ROS image capture of PANC-1 cell rupture

**Fig.S3.** Time-resolved fluorescence characterization of AV-EVLPs internalization in PANC-1 cells

**Fig.S4.** Analysis of mean fluorescence intensity *in vitro*

**Fig.S5.** Histological analysis was performed to evaluate the toxicity of EV-U. H&E-stained sections of major organs were obtained from tumor-bearing mice treated with saline, EV-U (2mg/ml), EV-U (4mg/ml), and Gemcitabine at the end of the experiment. Scale bar=100 μm.

**Fig.S6.** Long-time storage stability of protein in EV-U and EV-P when kept at –80°C for 1, 15, 30, 180 and 360 days

**Fig.S7.** The *in vivo* uptake of EV-U in tumor-bearing mice. DIO (green, EV-U) and DAPI (blue, nuclei) staining of cells of tumor tissues from different treatment groups. Scale bar=100 μm.

**Table.S1.** The primer sequence of target gene.

**Table.S2.** Lipid distribution ratio for EV-U and EV-P

**Table.S3.** Characterization of chemical constituents in the fresh juice and AV-EVLPs by UHPLC–QTOF MS


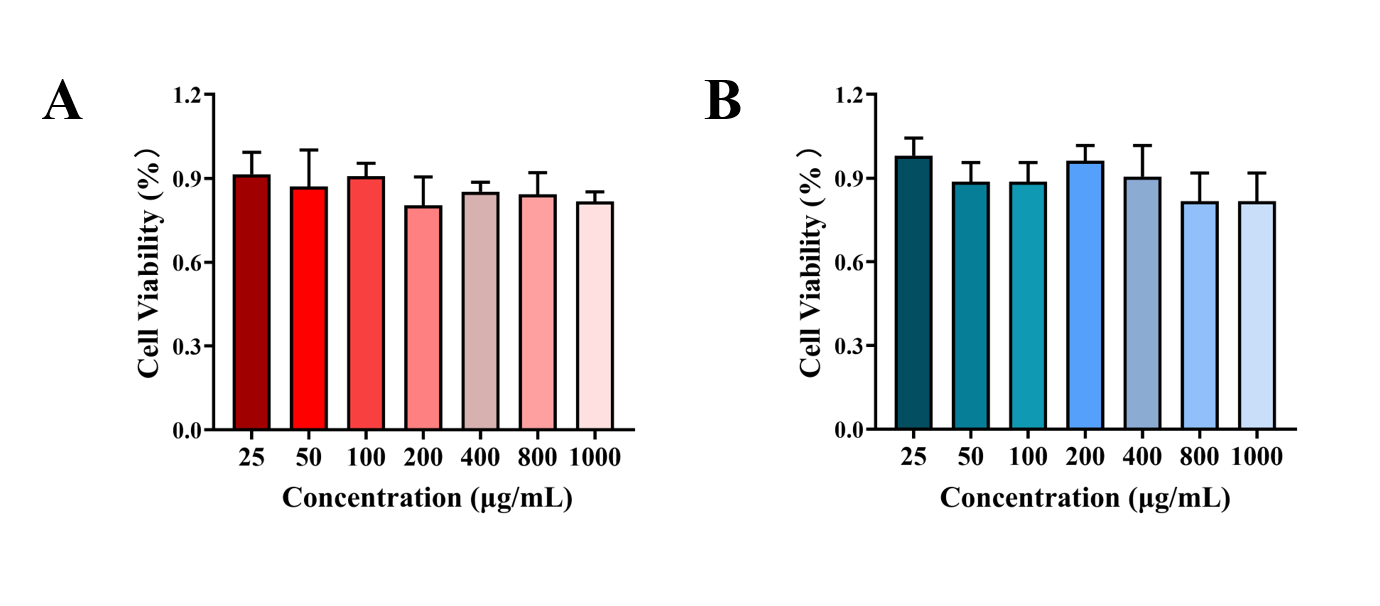


**Fig.S1.** The cell viability of H6C7 treated with different concentrations of EV-P (**A**) and EV-U (B) for 24 h evaluated using MTT assay.


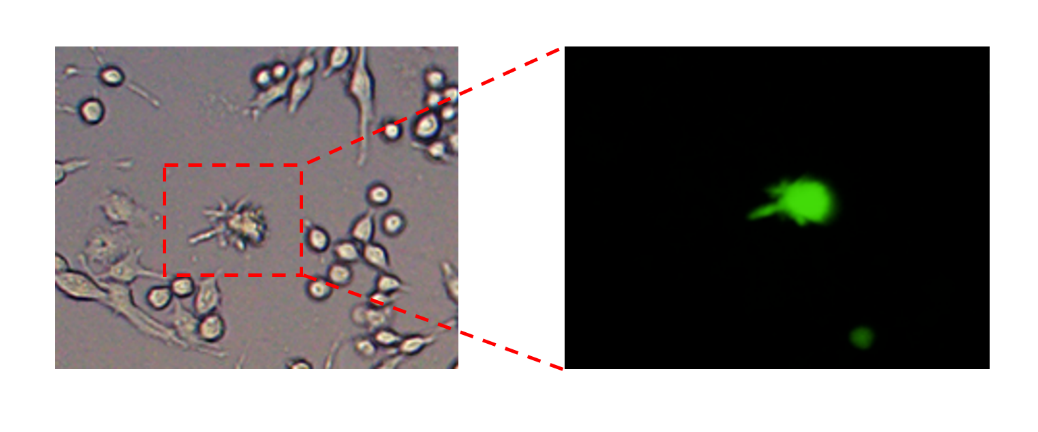


**Fig.S2.** ROS image capture of PANC-1 cell rupture


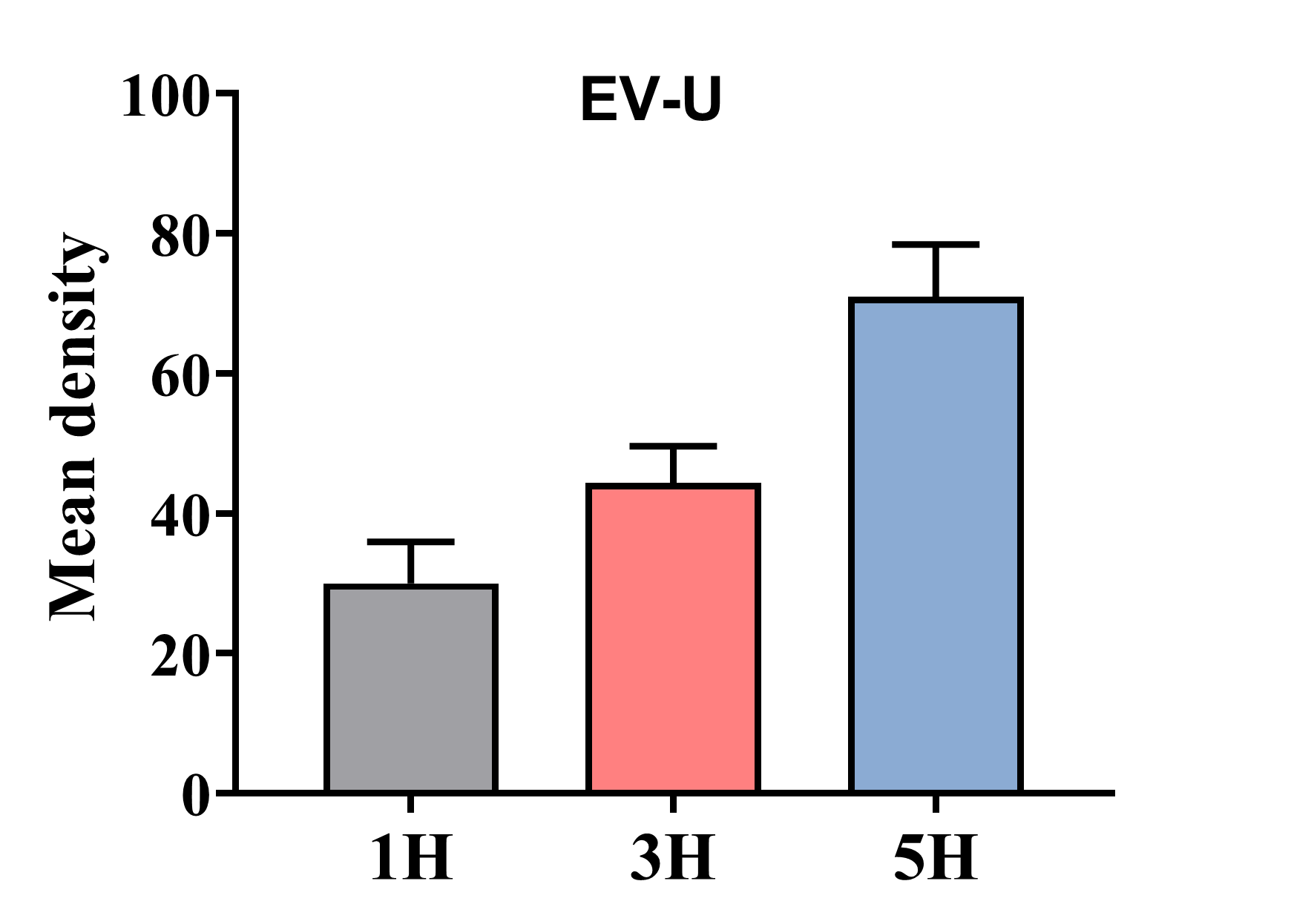

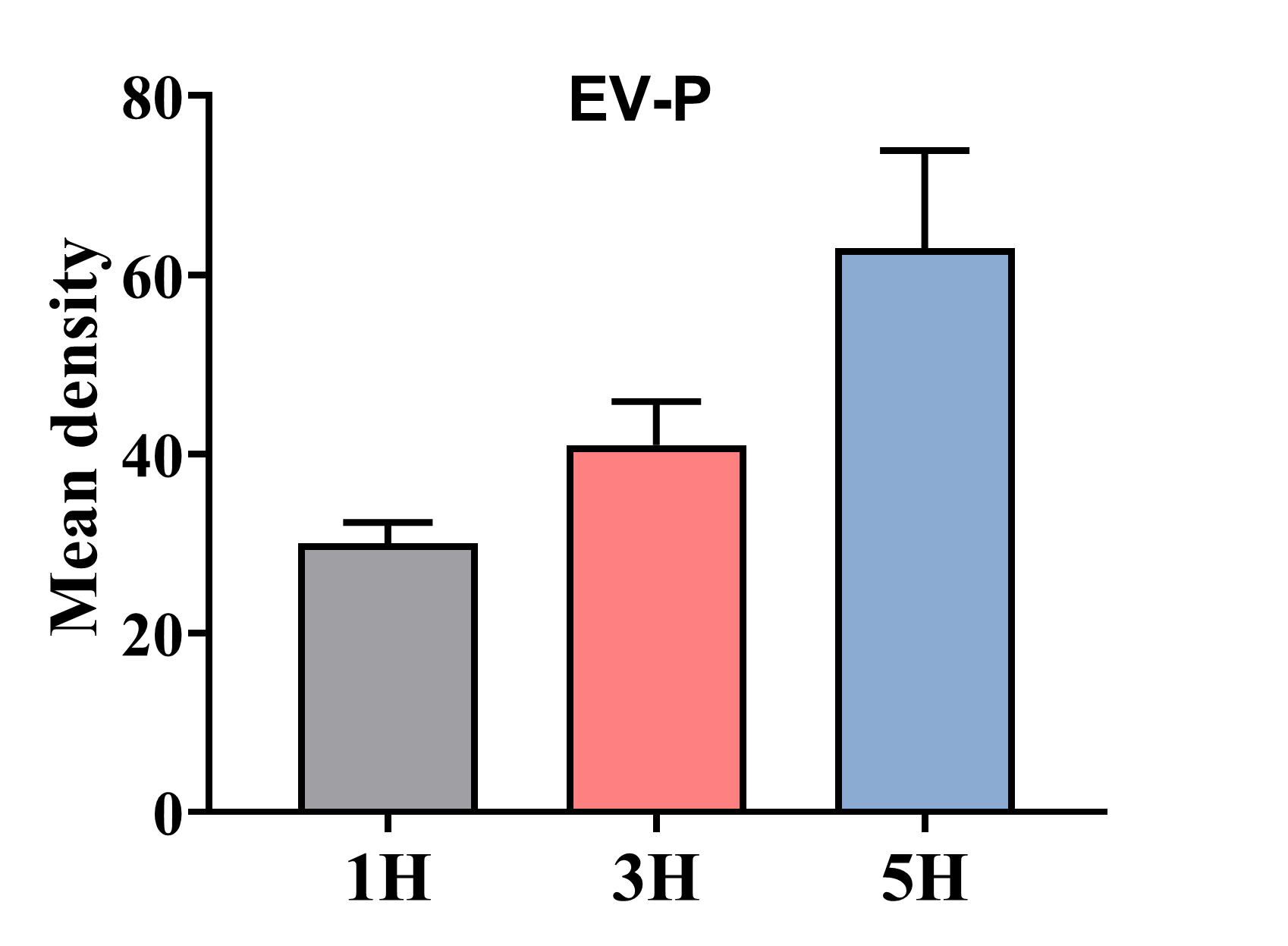


**Fig.S3.** Time-resolved fluorescence characterization of AV-EVLPs internalization in PANC-1 cells


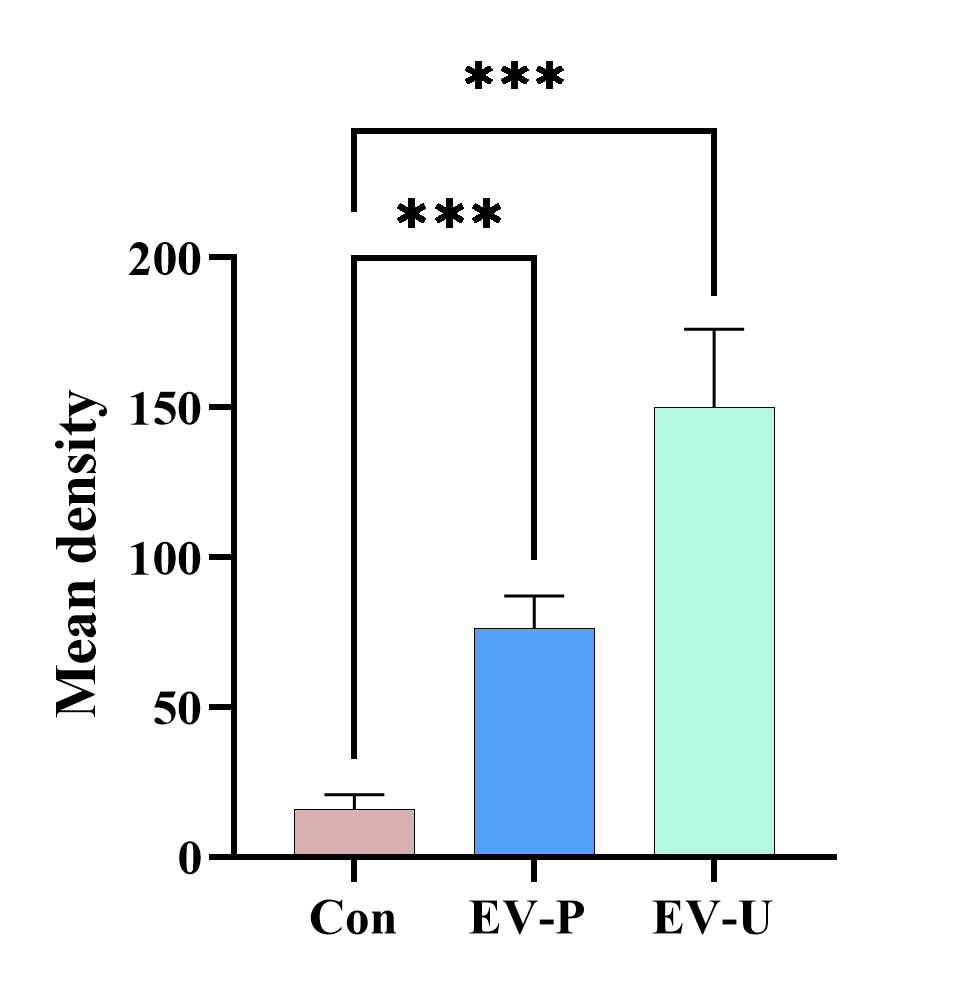
**Fig.S4.** Analysis of mean fluorescence intensity *in vitro*


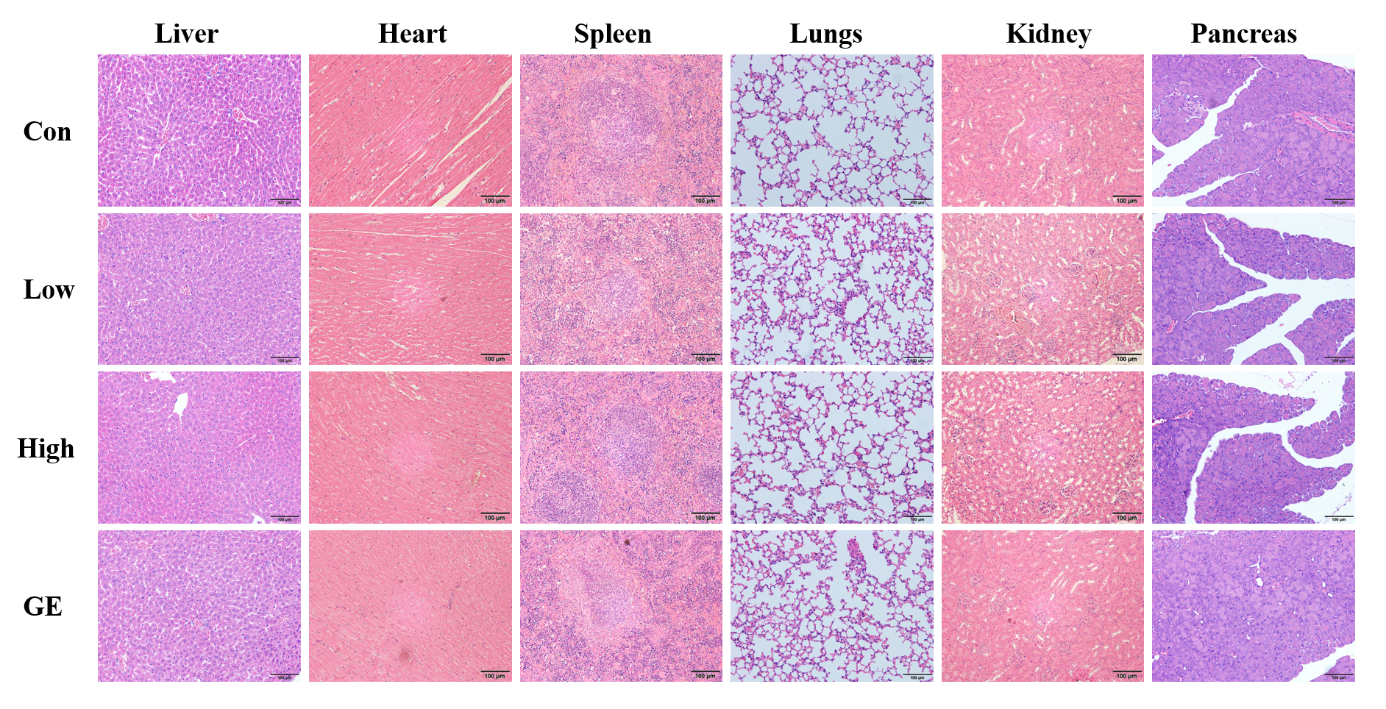


**Fig.S5.** Histological analysis was performed to evaluate the toxicity of EV-U. H&E-stained sections of major organs were obtained from tumor-bearing mice treated with saline, EV-U (2mg/ml), EV-U (4mg/ml), and Gemcitabine at the end of the experiment. Scale bar=100 μm.

**
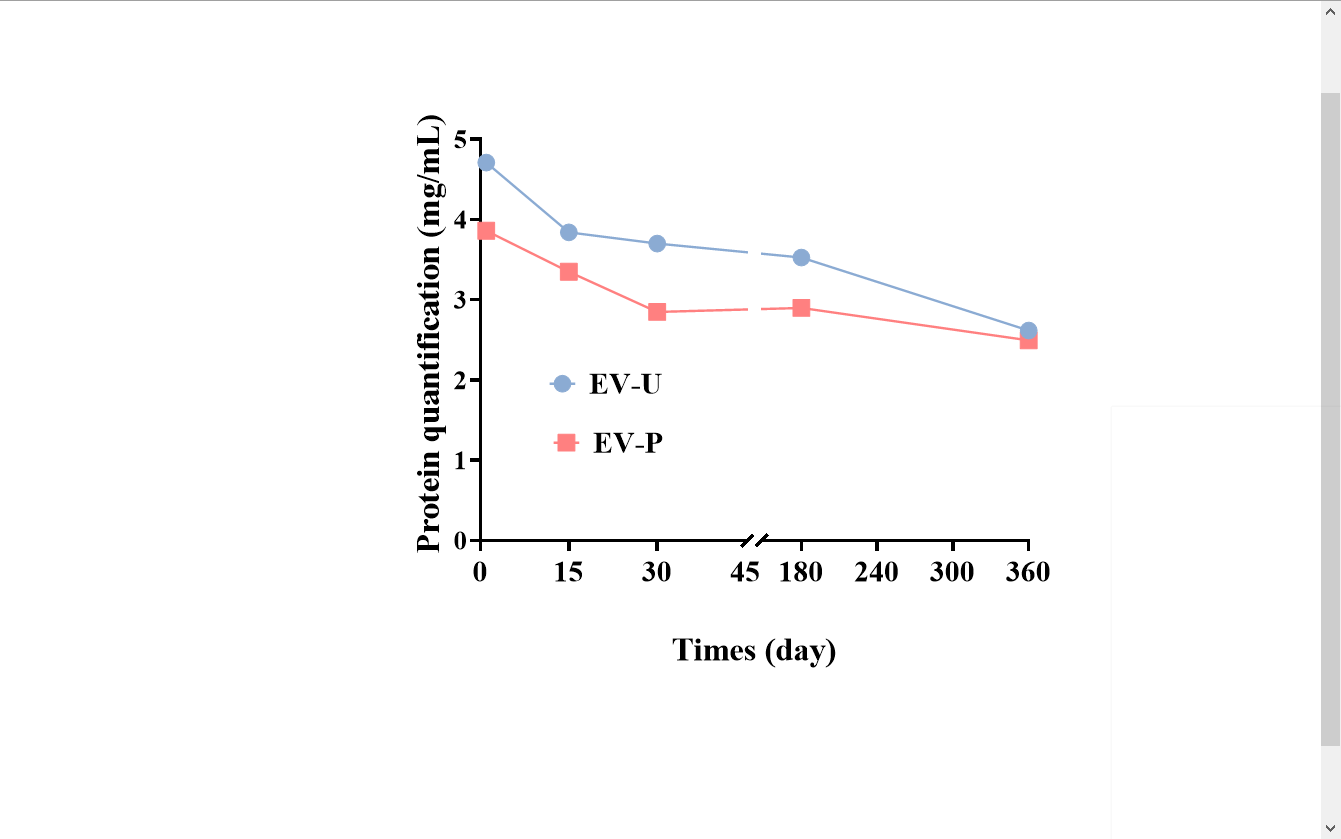
**

**Fig.S6.** Long-time storage stability of protein in EV-U and EV-P when kept at –80°C for 1, 15, 30, 180 and 360 days

**
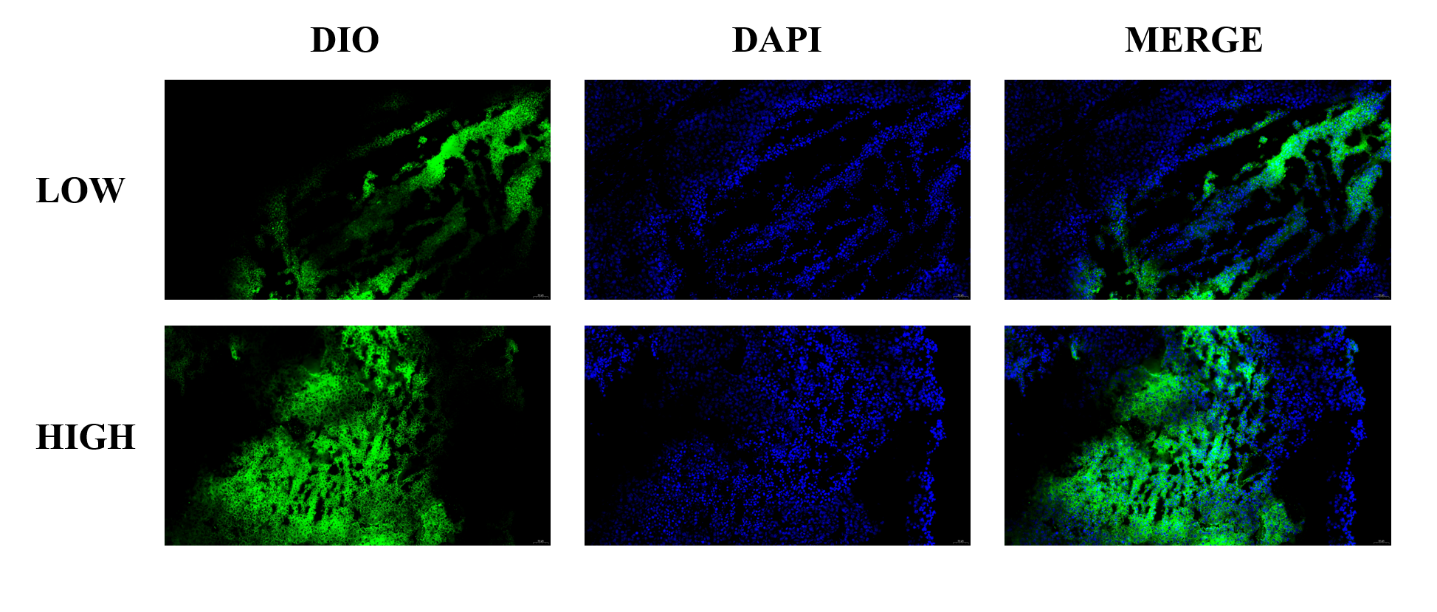
**

**Fig.S7.** The in vivo uptake of EV-U in tumor-bearing mice.

DIO (green, EV-U) and DAPI (blue, nuclei) staining of cells of tumor tissues from different treatment groups. Scale bar=100 μm

**Table.S1.** The primer sequence of target gene.

**mRNA Forward primer Reverse primer**

Caspase-1 CTATGGACAAGGCACGGGAC TCAGCTGATGGAGCTGATTGA

Caspase-3 AGCTTGGAACGGTACGCTAA GAGTCCACTGACTTGCTCCC

Caspase-7 GCCGTGGGAACGATGACC TTGGCGTCAACTCCGTCTTC

Caspase-9 GCCAGATGCTGTCCCCTATC AGCATTGGCAACCTGGGAAG

Gasdermin E AGGTCAGCAACCAGAAGCTC TGGCATTATCAGGCATTTCTGC

Gasdermin D AGTGCTCCAGAACCAGAACCG TCTGCCCTGAATGTTCCCATC

**Table.S2.** Lipid distribution ratio for EV-U and EV-P

**Lipid Class EV-P EV-U**

LysoPE 22 26

LysoPC 21 22

SM 24 40

PC 25 52

PE 17 33

CER 18 22

CE 6 9

DAG 36 37

FFA 32 32

PG 9 8

LysoPI 8 7

PI 3 3

PS 4 3

CHO 4 0

PA 4 0

**Supplemental Table S1 Characterization of chemical constituents in the fresh juice and** EVLPs **from Aloe by UHPLC–QTOF MS**

Peak t_R_ Identification Formula Quasi-molecular ion Observed mass Calculated mass ppm Fresh EV-P EV-U

No (min) (Da) (Da)

1 1.722 8-C-glucosyl-7-O-(S)-methyl- aloesol C_20_H_26_O_9_ [M+Cl]^—^ 445.1376 445.1271 -23.59 + / /

[M+HCOO]^—^ 455.1640 455.1559 -17.80

[M+CH3COO]^—^ 469.1697 469.1715 3.84

2 2.451 6'-O-(E)-coumaroylaloin A C_15_H_18_O_9_ [M+CH3COO]^—^ 401.1105 401.1089 -3.99 + / /

3 2.484 3'-O-β-D-glucopyranosyl-3R-feralolide C_27_H_30_O_17_ [M-H]^—^ 625.1403 625.1410 1.12 + / /

4 2.501 7-hydroxy-5-methyl-2-(2-oxopropyl)-8 C_19_H_22_O_9_ [M-H]^—^ 393.1227 393.1191 -9.16 + + +

-[(2S,3R,4R,5S,6R)-3,4,5-trihydroxy-6- [M+Cl]^—^ 429.0955 429.0958 0.70

(hydroxymethyl)oxan-2-yl] chromen-4-one

5 2.517 chrysophanol-1-O-β-D-glucoside C_21_H_20_O_9_ [M+Cl]^—^ 451.0764 451.0801 8.20 + + +

[M+HCOO]^—^ 461.1087 461.1089 0.43

6 2.931 chlorogenic acid C_16_H_18_O_9_ [M-H]^—^ 353.0894 353.0878 -4.53 + + +

[M+HCOO]^—^ 399.0896 399.0933 9.27

[M+CH3COO]^—^ 413.1099 413.1089 -2.42

7 3.279 (2'R)-8-C-glucosylaloesol C_19_H_24_O_9_ [M-H]^—^ 395.1388 395.1348 -10.12 + + +

[M+Cl]^—^ 431.1146 431.1114 -7.42

[M+CH3COO]^—^ 455.1579 455.1559 -4.39

8 3.279 8-(β-L-rhamnopyranosybxy)-3-(β-D)-xylopyranosyl C_24_H_30_O_12_ [M+HCOO]^—^ 555.1738 555.1719 -3.42 + / /

oxymethyl) napthalenol

9 3.227 8-C-glucosyl-7-O-(S)-methyl- aloesol C_20_H_26_O_9_ [M-H]^—^ 409.1491 409.1504 3.18 + + +

[M+Cl]^—^ 445.1289 445.1271 -4.04

[M+HCOO]^—^ 455.1579 455.1559 -4.39

10 3.494 rutin C_27_H_30_O_16_ [M-H]^—^ 609.1485 609.1461 -3.94 + + +

[M+Cl]^—^ 645.1236 645.1228 -1.24

[M+CH3COO]^—^ 669.1834 669.1672 -24.21

11 3.511 isoquercitrin C_21_H_20_O_12_ [M-H]^—^ 463.0901 463.0882 -4.10 + + +

12 3.577 5-((S)-2'-oxo-4'-Hydroxypentyl)-2-methoxychromone C_15_H_16_O_6_ [M+HCOO]^—^ 337.0939 337.0929 -2.97 + + +

13 3.610 gardoside C_16_H_22_O_10_ [M+CH3COO]^—^ 433.1376 433.1351 -5.77 + / /

14 3.610 8-epiapodantheroside C_17_H_24_O_10_ [M-H]^—^ 387.1265 387.1297 8.27 + / /

[M+Cl]^—^ 423.1094 423.1063 -7.33

[M+HCOO]^—^ 433.1376 433.1351 -5.77

15 3.859 7-O-Methylaloeresin A C_20_H_24_O_9_ [M-H]^—^ 407.1338 407.1348 2.46 + + +

16 3.875 3,5-di-O-caffeoyl-4-O-(3-hydroxy-3-methyl)- C_31_H_32_O_16_ [M-H]^—^ 659.1604 659.1618 2.12 + + +

glutaroylquinic acid [M+Cl]^—^ 695.1360 695.1384 3.45

17 3.925 5, 7, 3',4'-tetrahydroxy-6,8-dimethoxy flavone C_17_H_14_O_8_ [M+HCOO]^—^ 391.0666 391.0671 1.28 + / /

18 3.958 aloeresin C C_34_H_38_O_16_ [M-H]^—^ 701.2103 701.2087 -2.28 + / /

[M+Cl]^—^ 737.2021 737.1854 -22.65

19 4.107 nicotiflorin C_27_H_30_O_15_ [M-H]^—^ 593.1552 593.1512 -6.74 + + +

[M+Cl]^—^ 629.1286 629.1279 -1.11

20 4.256 6'''-Sinapoylspinosin C_39_H_42_O_19_ [M-H]^—^ 813.2252 813.2248 -0.49 + / /

[M+Cl]^—^ 849.2450 849.2014 -51.34

21 4.341 plicataloside C_23_H_30_O_13_ [M+Cl]^—^ 549.1375 549.138 0.91 / + +

22 4.521 2'-O-(4-Hydroxy-3-methoxy-E-cinnamoyl) C_30_H_32_O_12_ [M-H]^—^ 583.1978 583.1821 -26.92 + / /

aloesin 7-methyl ether [M+HCOO]^—^ 629.1871 629.1876 0.79

[M+CH3COO]^—^ 643.1995 643.2032 5.75

23 4.621 10-Hydroxyaloin B C_21_H_22_O_10_ [M+Cl]^—^ 469.0867 469.0907 8.53 + + +

[M+CH3COO]^—^ 493.1384 493.1351 -6.69

24 4.654 4'-O-glucosyl-isoaloeresin DI C_35_H_42_O_16_ [M-H]^—^ 717.2506 717.2400 -14.78 + + +

[M+Cl]^—^ 753.2171 753.2167 -0.53

[M+HCOO]^—^ 763.2461 763.2455 -0.79

25 4.655 sinapyglucoside C_17_H_22_O_10_ [M-H]^—^ 385.1152 385.114 -3.12 / + +

26 4.770 (2'R)-8-C-glucosylaloesol C_19_H_24_O_9_ [M-H]^—^ 395.1371 395.1348 -5.82 + / /

[M+Cl]^—^ 431.1171 431.1114 -13.22

**Supplemental Table S1 Characterization of chemical constituents in the fresh juice and** EVLPs **from Aloe by UHPLC–QTOF MS (*Continued*)**

Peak t_R_ Identification Formula Quasi-molecular ion Observed mass Calculated mass ppm Fresh EV-P EV-U

No (min) (Da) (Da)

27 4.803 asminoside B C_16_H_26_O_8_ [M-H]^—^ 345.1532 345.1555 6.66 + + /

[M+Cl]^—^ 381.1388 381.1322 -17.32

[M+HCOO]^—^ 391.1666 391.1610 -14.32

[M+CH3COO]^—^ 405.1761 405.1766 1.23

28 4.821 epicatechin gallate C_22_H_18_O_10_ [M+HCOO]^—^ 487.0869 487.0882 2.67 / + +

29 4.853 10-Hydroxyaloin A C_21_H_22_O_10_ [M-H]^—^ 433.1174 433.1140 -7.85 + + +

[M+Cl]^—^ 469.0936 469.0907 -6.18

[M+HCOO]^—^ 479.1275 479.1195 -16.70

[M+CH3COO]^—^ 493.1325 493.1351 5.27

30 4.937 aloenin-2'-p-coumaroyl ester C_28_H_28_O_12_ [M-H]^—^ 555.1509 555.1508 -0.18 / + +

31 4.937 aloe-emodin-8-O-beta-D-glucopyranoside C_21_H_20_O_10_ [M-H]^—^ 431.0976 431.0984 1.86 + + +

32 5.118 homonataloin C_21_H_22_O_10_ [M-H]^—^ 433.1170 433.1140 -6.93 + + +

[M+Cl]^—^ 469.0929 469.0907 -4.69

[M+CH3COO]^—^ 493.1418 493.1351 -13.59

33 5.118 isoquercitrin C_21_H_20_O_12_ [M-H]^—^ 463.0913 463.0882 -6.69 + + +

34 5.383 6'-O-trans-coumaroyl geniposidic acid C_25_H_28_O_12_ [M-H]^—^ 519.1603 519.1508 -18.30 + + +

[M+CH3COO]^—^ 579.1734 579.1719 -2.59

35 5.383 6'-O-trans-sinapoyl gardoside C_27_H_32_O_14_ [M-H]^—^ 579.1734 579.1719 -2.59 + + +

[M+Cl]^—^ 615.1511 615.1486 -4.06

[M+HCOO]^—^ 625.1824 625.1774 -8.00

36 5.383 6'-O-trans-coumaroyl geniposide C_26_H_30_O_12_ [M+Cl]^—^ 569.1493 569.1431 -10.89 + / /

[M+HCOO]^—^ 579.1736 579.1719 -2.94

37 5.466 7-hydroxy-5-methyl-2-(2-oxopropyl)-8- C_19_H_22_O_9_ [M-H]^—^ 393.1207 393.1191 -4.07 + + +

[(2S,3R,4R,5S,6R)-3,4,5-trihydroxy-6-

(hydroxymethyl)oxan-2-yl]chromen-4-one

38 5.532 iso-homonataloin C_21_H_22_O_10_ [M-H]^—^ 433.1186 433.1140 -10.62 + / /

[M+Cl]^—^ 469.0922 469.0907 -3.20

[M+HCOO]^—^ 479.1226 479.1195 -6.47

39 5.565 barbaloin A C_28_H_28_O_11_ [M-H]^—^ 539.1583 539.1559 -4.45 + + +

[M+Cl]^—^ 575.1612 575.1632 3.48

[M+HCOO]^—^ 585.1622 585.1614 -1.37

[M+CH3COO]^—^ 599.1847 599.1770 -12.85

40 5.731 rabaichromone C_29_H_32_O_12_ [M-H]^—^ 571.1851 571.1821 -5.25 + + +

[M+Cl]^—^ 607.1625 607.1588 -6.09

[M+CH3COO]^—^ 631.2216 631.2032 -29.15

41 5.749 7-Oethylaloere sinA C_29_H_30_O_11_ [M+HCOO]^—^ 599.1762 599.177 1.34 / + +

42 5.813 6'-O-trans-coumaroyl geniposide C_26_H_30_O_12_ [M-H]^—^ 533.1748 533.1664 -15.75 + / /

[M+Cl]^—^ 569.1397 569.1431 5.97

[M+HCOO]^—^ 579.1743 579.1719 -4.14

[M+CH3COO]^—^ 593.1969 593.1876 -15.68

43 5.996 aloin C_21_H_22_O_9_ [M-H]^—^ 417.1208 417.1191 -4.08 + / /

44 6.029 aloinoside A C_27_H_32_O_13_ [M-H]^—^ 563.1823 563.1770 -9.41 + / /

[M+Cl]^—^ 599.1494 599.1537 7.18

[M+HCOO]^—^ 609.1792 609.1825 5.42

[M+CH3COO]^—^ 623.1976 623.1981 0.80

45 6.112 aloe-emodin C_15_H_10_O_5_ [M+Cl]^—^ 305.0237 305.0222 -4.92 + / /

[M+HCOO]^—^ 315.0504 315.0510 1.90

[M+CH3COO]^—^ 329.0653 329.0667 4.25

46 6.161 asminoside B C_16_H_26_O_8_ [M-H]^—^ 345.1521 345.1555 9.85 + / /

[M+Cl]^—^ 381.1413 381.1322 -23.88

[M+HCOO]^—^ 391.1591 391.1610 4.86

[M+CH3COO]^—^ 405.1757 405.1766 2.22

47 6.194 jasminodiol C_10_H_16_O_3_ [M+CH3COO]^—^ 243.1237 243.1238 0.41 + + +

**Supplemental Table S1 Characterization of chemical constituents in the fresh juice and** AV-EVLPs **from Aloe by UHPLC–QTOF MS (*Continued*)**

Peak t_R_ Identification Formula Quasi-molecular ion Observed mass Calculated mass ppm Fresh EV-P EV-U

No (min)

48 6.261 aloin-A C_21_H_22_O_9_ [M-H]^—^ 417.1225 417.1191 -8.15 + + +

[M+Cl]^—^ 453.0980 453.0958 -4.86

[M+HCOO]^—^ 463.1259 463.1246 -2.81

49 6.343 (E)-2-acetonyl-8-(2'-O-caffeoyl)-β-D)-glucopyranosyl- C_29_H_30_O_12_ [M-H]^—^ 569.1669 569.1664 -0.88 + + +

7-methoxy-5-methylchromone [M+Cl]^—^ 605.1504 605.1431 -12.06

[M+HCOO]^—^ 615.1874 615.1719 -25.20

[M+CH3COO]^—^ 629.1899 629.1876 -3.66

50 6.343 aloeresin D C_29_H_32_O_11_ [M-H]^—^ 555.1911 555.1872 -7.02 + + +

[M+Cl]^—^ 591.1640 591.1639 -0.17

51 6.509 7-Oethylaloere sinA C_29_H_30_O_11_ [M-H]^—^ 553.1736 553.1715 -3.80 + + +

[M+CH3COO]^—^ 613.1881 613.1927 7.50

52 6.559 aloin-B C_21_H_22_O_9_ [M-H]^—^ 417.1229 417.1191 -9.11 + + +

[M+Cl]^—^ 453.0998 453.0958 -8.83

[M+HCOO]^—^ 463.1280 463.1264 -3.45

53 6.691 ixoroside C_16_H_24_O_9_ [M+CH3COO]^—^ 419.1576 419.1559 -4.06 + / /

54 6.691 jasminoside T C_21_H_34_O_11_ [M+Cl]^—^ 497.1847 497.1795 -10.46 + / /

[M+HCOO]^—^ 507.2162 507.2083 -15.58

[M+CH3COO]^—^ 521.2222 521.2240 3.45

55 6.907 crocusatin-C C_10_H_16_O_2_ [M+HCOO]^—^ 213.1136 213.1132 -1.88 + + +

[M+CH3COO]^—^ 227.1292 227.1289 -1.32

56 7.172 cinnamic acid C_9_H_8_O_2_ [M+HCOO]^—^ 193.0519 193.0506 -6.73 + / /

[M+CH3COO]^—^ 207.0648 207.0663 7.24

57 7.254 6'-O-sinapoylgeniposide C_28_H_34_O_14_ [M-H]^—^ 593.1871 593.1876 0.84 + / /

[M+Cl]^—^ 629.1702 629.1643 -9.38

[M+HCOO]^—^ 639.2045 639.1931 -17.83

[M+CH3COO]^—^ 653.2024 653.2087 9.64

58 7.538 quercetin C_15_H_10_O_7_ [M-H]^—^ 301.0363 301.0354 -2.99 / + +

[M+Cl]^—^ 337.0104 337.0121 5.04

59 7.836 bruceantarin C_28_H_30_O_11_ [M+CH3COO]^—^ 601.1889 601.1927 6.32 / + +

60 7.851 physcion C_16_H_12_O_5_ [M+CH3COO]^—^ 343.0843 343.0823 -5.83 + + +

61 8.397 microdontin B C_30_H_28_O_11_ [M-H]^—^ 563.1578 563.1559 -3.37 + + +

[M+Cl]^—^ 599.1342 599.1326 -2.67

[M+HCOO]^—^ 609.1754 609.1614 -22.98

[M+CH3COO]^—^ 623.1877 623.1770 -17.17

62 8.480 aloe-emodin C_15_H_10_O_5_ [M-H]^—^ 269.0434 269.0455 7.81 + / +

63 8.531 (E)-2-Acetonyl-8-[(2''-O-cinnamoyl)-β-d- C_29_H_30_O_10_ [M-H]^—^ 537.1755 537.1766 2.05 / + +

glucopyranosyl-7-methoxy-5-methylchromone [M+Cl]^—^ 573.1522 573.1533 1.92

64 8.580 microdontin B C_30_H_28_O_11_ [M-H]^—^ 563.1581 563.1559 -3.91 + + +

[M+Cl]^—^ 599.1351 599.1326 -4.17

[M+HCOO]^—^ 609.1562 609.1614 8.54

[M+CH3COO]^—^ 623.1770 623.1770 0.00

65 9.573 gadenone C_12_H_20_O_3_ [M-H]^—^ 211.1332 211.1340 3.79 + / /

[M+Cl]^—^ 247.1096 247.1106 4.05

[M+HCOO]^—^ 257.1427 257.1394 -12.83

[M+CH3COO]^—^ 271.1542 271.1551 3.32

66 10.037 Dehydroandrographolide C_20_H_28_O_4_ [M+Cl]^—^ 367.1653 367.1682 7.90 + / /

[M+HCOO]^—^ 377.1921 377.1903 -4.77

67 10.402 gallic acid C_7_H_6_O_5_ [M-H]^—^ 169.0150 169.0142 -4.73 + + +
